# Chick Hippocampal Formation Displays Subdivision- and Layer-Selective Expression Patterns of Serotonin Receptor Subfamily Genes

**DOI:** 10.3389/fphys.2022.882633

**Published:** 2022-04-08

**Authors:** Toshiyuki Fujita, Naoya Aoki, Chihiro Mori, Eiko Fujita, Toshiya Matsushima, Koichi J. Homma, Shinji Yamaguchi

**Affiliations:** ^1^ Department of Biological Sciences, Faculty of Pharmaceutical Sciences, Teikyo University, Tokyo, Japan; ^2^ Department of Molecular Biology, Faculty of Pharmaceutical Sciences, Teikyo University, Tokyo, Japan; ^3^ Department of Biology, Faculty of Science, Hokkaido University, Sapporo, Japan

**Keywords:** chick, hippocampal formation, serotonin receptor, subdivision, layer

## Abstract

Hippocampal formation (HF) plays a key role in cognitive and emotional processing in mammals. In HF neural circuits, serotonin receptors (5-HTRs) modulate functions related to cognition and emotion. To understand the phylogenetic continuity of the neural basis for cognition and emotion, it is important to identify the neural circuits that regulate cognitive and emotional processing in animals. In birds, HF has been shown to be related to cognitive functions and emotion-related behaviors. However, details regarding the distribution of 5-HTRs in the avian brain are very sparse, and 5-HTRs, which are potentially involved in cognitive functions and emotion-related behaviors, are poorly understood. Previously, we showed that *5-HTR1B* and *5-HTR3A* were expressed in chick HF. To identify additional 5-HTRs that are potentially involved in cognitive and emotional functions in avian HF, we selected the chick orthologs of *5-HTR1D*, *5-HTR1E*, *5-HTR1F*, *5-HTR2B*, *5-HTR5A*, and *5-HTR7* and performed *in situ* hybridization in the chick telencephalon. We found that *5-HTR1D, 5-HTR1E, 5-HTR5A*, and *5-HTR7* were expressed in the chick HF, especially *5-HTR1D* and *5-HTR1E*, which showed subdivision- and layer-selective expression patterns, suggesting that the characteristic 5-HT regulation is involved in cognitive functions and emotion-related behaviors in these HF regions. These findings can facilitate the understanding of serotonin regulation in avian HF and the correspondence between the HF subdivisions of birds and mammals.

## Introduction

The modulation of various neural functions by serotonin (5-hydroxytryptamine, 5-HT) is phylogenetically conserved (Marin et al., 2020). In particular, the association of the 5-HT system with cognition, behavior, and emotion is evolutionarily conserved in the animal kingdom (Kandel et al., 2000; Bacque-Cazenave et al., 2020). In mammals, processing of both cognition and emotion have been shown to involve the hippocampal formation (HF) (Fanselow and Dong, 2010; Anacker and Hen, 2017). The macrohistological features of HF are well-conserved in all mammals. More specifically, HF consists of similarly convoluted and interlocked 3-layered subdivisions: the dentate gyrus (DG), Ammon’s horns or Cornu ammonis (CA) fields 1 to 3 (CA1 to CA3), and the subiculum (Hevner, 2016; Medina et al., 2017b). Information flow in the mammalian HF was described as a “trisynaptic circuit”, in which the entorhinal cortex (EC) projects to the DG, DG provides the projection to CA3 known as “mossy fiber”, and CA3 relays it to CA1 (Sloviter and Lomo, 2012). To understand the phylogenetic continuity of the neural basis for cognition and emotion, it is essential to reveal the neural mechanisms in HF that process cognitive and emotional behaviors in nonmammalian animals. Birds are well-fitted model animals for understanding the evolutionary continuity of the neural basis of cognition and emotion (Rosa Salva et al., 2015; Papini et al., 2019). For instance, the polymorphism of the 5-HT transporter gene, which affects the levels of gene expression, has been suggested to modulate fear-related behavior in chickens (Krause et al*.*, 2017; Phi Van et al., 2018; Krause et al., 2019). In addition, hippocampal formation in birds (HF, hippocampus proper (Hp), and area parahippocampalis (APH)) has been shown to be related to cognitive functions, such as spatial navigation (Colombo and Broadbent, 2000; Matsushima et al., 2003; Bingman, et al., 2005; Mayer, et al., 2016; Sherry, et al., 2017; Payne, et al., 2021), and controlling the stress response (Smulders, 2017; Smulders, 2021) and emotions, such as anxiety-like behavior (Mayer, et al., 2018; Morandi-Raikov and Mayer, 2020; Parada, et al., 2021). However, the neural circuits that control these behaviors and 5-HT regulation in the avian HF are largely unknown.

The avian HF is homologous to that of mammals (Reiner, et al., 2004; Herold, et al., 2015; Striedter, 2016). The ancient origin of HF in the amniote was supported by both recent large-scale and single-cell transcriptome studies (Belgard, et al., 2013; Tosches, et al., 2018). However, the avian HF is composed of a layered arrangement of densely packed neurons with poorly defined boundaries, whereas the mammalian HF has a clear laminar organization (Atoji and Wild, 2006; Herold, et al., 2015; Striedter, 2016). The existence of many subdivisions in the avian HF has been proposed to arise from multiple aspects, such as developmental origin, connectivity, histochemistry, and immunohistochemistry (Kuenzel and Masson, 1988; Atoji and Wild, 2004; Atoji and Wild, 2006; Suarez, et al., 2006; Puelles, et al., 2007; Gupta, et al., 2012; Herold, et al., 2014; Abellan, et al., 2014; Atoji, et al., 2016; Medinia, et al., 2017b). However, to date, a one-to-one correspondence of subdivisions between the avian and mammalian HF has not been established, especially owing to the controversy regarding its homology with the mammalian Hp (dentate gyrus and Ammon’s horn) (Atoji and Wild, 2004; Atoji and Wild, 2006; Kempermann, 2012; Herold, et al., 2014; Abellan, et al., 2014; Streidter, 2016; Hevner, 2016; Atoji et al., 2016; Medina, et al., 2017b).

In mammals, various types of the 5-HT receptor (5-HTR) subfamily genes are expressed in the HF and are thought to play important roles in cognitive and emotional functions (Tanaka, et al., 2012; Strac et al., 2016; Zmudzka, et al., 2018; O’Leary, et al., 2020; Vilaro, et al., 2020). In our previous study, we pointed out that *5-HTR1B* and *5-HTR3A* were expressed in chick HF in a clear and characteristic manner (Fujita, et al., 2020). In the present study, we performed a detailed analysis and determined the subdivision of HF in which *5-HTR1B* and *5-HTR3A* are expressed. To comprehensively identify more 5-HTRs that are potentially involved in cognitive and emotional functions in avian HF, we investigated the expression of 5-HTR subfamily genes that were not analyzed in our previous study (Fujita, et al., 2020). We selected the chick orthologues of *5-HTR1D*, *5-HTR1E*, *5-HTR1F*, *5-HTR2B*, *5-HTR5A*, and *5-HTR7* and found that *5-HTR1D, 5-HTR1E, 5-HTR5A*, and *5-HTR7* were expressed in the chick HF. Among them, *5-HTR1D* and *5-HTR1E* showed subdivision- and layer-selective expression patterns, suggesting a characteristic 5-HT regulation in these regions. Our findings can be used as a basis for understanding 5-HT regulation in avian HF and the correspondence between the HF subdivisions of birds and mammals.

## Materials and Methods

### Animals

Fertilized eggs of domestic chicks (*Gallus domesticus*, Cobb strain) were purchased from a local dealer (3-M, Aichi, Japan) and incubated at Teikyo University (Kaga, Itabashi-ku, Tokyo, Japan). Animal experiments were performed as previously described (Yamaguchi et al., 2008a; 2008b). Newly hatched chicks (P0) were transferred to dark plastic enclosures in a dark, warm cage at 30°C for 1 day (P1). We used seven chicks for *5-HTR1D* probes, nine for *5-HTR1E*, eight for 5-*HTR1F*, seven for 5-*HTR2B*, six for 5-*HTR5A*, six for 5-*HTR7*, three for 5-*HTR3A*, five for 5-*HTR1B*, and six for *lymphoid enhancer factor 1* (*LEF1*) (Supplementary Table S1). As for *5-HTR3A* and *5-HTR1B*, we have already investigated the expression of *5-HTR3A* and *5-HTR1B* throughout the entire brain in our previous study. In this study we focused on the expression of *5-HTR3A* and *5-HTR1B* in the HF. All procedures were reviewed and approved by the Committee on Animal Experiments of Teikyo University and were conducted in accordance with the guidelines of the national regulations for animal welfare in Japan.

### Tissue Preparation

P1 chicks were anesthetized by intraperitoneal injection (0.40 ml/individual) of a 1:1 solution of ketamine (10 mg/ml, Ketalar-10, Sankyo Co., Tokyo, Japan) and xylazine (2 mg/ml, Sigma, St. Louis, MO, United States). For brain fixation, the anesthetized chicks were transcardially perfused with 4% paraformaldehyde in 0.1 M phosphate buffered saline (pH 7.5, PFA-PBS). Whole brain specimens were dissected and immediately immersed in PFA-PBS for 1 or 2 days at 4°C. For cryoprotection, fixed brain samples were placed in an 18% sucrose/PFA-PBS solution for 2 days at 4°C. Subsequently, brains with sucrose substitution were embedded in Tissue-Tek OCT compound (Sakura Finetechnical, Tokyo, Japan), frozen immediately on dry ice, and stored at −80°C until sectioning. Frozen brain blocks were cut into 18 µm-thick sections using a cryostat (Leica CM3050S or Leica CM 1850, Leica Biosystems, Nußloch, Germany). Serial coronal sections were prepared at the level of A14.4–A4.4, corresponding to those of the atlas by Kuenzel and Masson (1988).

### cDNA Cloning

Total RNA was extracted from chick brains using the TRIzol reagent (Invitrogen, Carlsbad, CA, United States) and reverse-transcribed using the SuperScript III kit (Invitrogen) with an oligo (dT) primer, according to the manufacturer’s protocol. Reverse transcription polymerase chain reaction (RT-PCR) for the amplification of *LEF1* was performed using gene-specific primers: forward, 5′-GAT​CCC​CTT​CAA​GGA​CGA​AG-3′; and reverse, 5′-GCC​AAG​AGG​TGG​TGT​TAT​CTG-3′. PCR products were subcloned into the pGEM-T easy vector (Promega, Madison, WI, United States), the sequence of which was validated using Sanger sequencing. For 5-*HTR1B, 5-HTR1D, 5-HTR1E, 5-HTR1F, 5-HTR2B*, *5-HTR3A*, *5-HTR5A*, and *5-HTR7* probes, we used previously generated plasmids (Fujita et al., 2020; Fujita et al., 2022).

### RNA Probe Preparations

Plasmids containing cDNA fragments for *5-HTR1B, 5-HTR1D, 5-HTR1E, 5-HTR1F, 5-HTR2B, 5-HTR3A, 5-HTR5A*, *5-HTR7*, and *LEF1* were amplified by PCR using the M13 primer pair. Amplicons containing T7 and SP6 promoter sites were purified using a PCR purification kit (Qiagen, Valencia, CA, United States). Digoxigenin (DIG)-labelled sense and antisense RNA probes were prepared by *in vitro* transcription using a DIG RNA labelling kit (Roche, Basel, Switzerland) according to the manufacturer’s protocol.

### *In situ* Hybridization

ISH experiments were performed as previously described in Fujita et al. (2019), with some modifications. Brain section specimens were refixed in 4% PFA-PBS, pretreated, and hybridized with DIG-labelled RNA probes at 70°C. After stringent washes with a series of saline-sodium citrate (SSC) buffers, hybridized probes were detected via immunohistochemical examination using an alkaline phosphatase-conjugated anti-DIG antibody (1:1,000; Roche). For signal visualization, a chromogenic reaction with a nitro blue tetrazolium/5-bromo-4-chloro-3-indolyl phosphate (NBT/BCIP) was performed at 25°C for the following durations: *5-HTR1D, 5-HTR1E, 5-HTR1F*, and *5-HTR2B*, 18–42.5 h; 5-*HTR5A*, 18.3–39.5 h; 5-*HTR7*, 19.5–39.8 h; 5-*HTR1B*, 18.2–39 h; 5-*HTR3A*, 19.5 h; and *LEF1*, 18–38.8 h. Sense probes were used as negative controls in every experiment.

### Image Acquisition and Data Processing

Bright-field images of whole sections on each slide glass were semiautomatically taken using the NanoZoomer 2.0 HT or NanoZoomer XR systems (Hamamatsu Photonics, Shizuoka, Japan). Microscopic fields of interest were cropped using the NDP.view2 software (ver. 2.7.25; Hamamatsu Photonics, Shizuoka, Japan). Cropped images were converted to 8-bit images and their brightness and contrast were adjusted using ImageJ (ver. 1.52a, National Institute of Health, Bethesda, MD, United States).

### Terminology of Avian Hippocampal Formation

**FIGURE 1:**
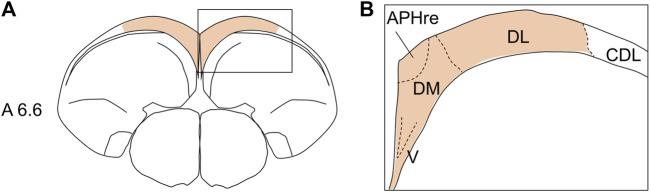
Position of chick HF and its subdivisions **(A)** diagram of the coronal section of the chick telencephalon showing the HF position **(A)**. Subdivisions of the chick HF **(B)**. See Table 1 for chick HF terminology. Regarding the HF range, to this day, no consensus or view for the inclusion of CDL exists (Puelles et al., 2007; Medina et al., 2017b). APHre, ectopic part of the rostral area parahippocampalis; CDL, corticoidea dorsolateralis; DL, dorsal lateral region of HF; DM, dorsal medial region of HF; V, V-shaped complex. Levels of sections were in accordance with those mentioned in Kuenzel and Masson’s atlas (Kuenzel and Masson, 1988).

All histological terminologies used in this study were based on the atlas of Kuenzel and Masson (1988) and the descriptions of the avian brain nomenclature consortium (Reiner et al., 2004), except for HF and its subdivisions. The subdivision schemes of avian HF were based on various aspects and species, such as tract tracing and Nissl staining in pigeons (Atoji and Wild, 2004; Atoji and Wild, 2006), histochemistry of neurotransmitter radioligands in pigeons (Herold, et al., 2014), immunohistochemistry of several neurochemical markers in developing chickens (Suarez, et al., 2006), and combinatorial expression patterns of morphogenetic genes in developing chickens (Abellan, et al., 2014). No uniform nomenclature has been applied across species (Herold, et al., 2015). In this study, the histological position of HF in chicks was based on the nomenclature set by Puelles et al*.* (2007). Based on previous studies (Kuenzel and Masson, 1988; Atoji and Wild, 2004; Atoji and Wild, 2006; Suarez, et al., 2006; Puelles, et al., 2007; Abellan, et al., 2014; Herold, et al., 2014), we used the following subdivision terms: V-shaped complex (V), dorsal medial region (DM), ectopic part of the rostral APH (APHre), and dorsal lateral region (DL) (Figure 1). The relationships between the terminologies of avian HF used in this study and those in previous studies are summarized in Table 1.

**TABLE 1:** Comparison of terminology regarding avian HF.

Chicken			Pigeon
This study, 2022	Kuenzel and Masson, (1988)	Suarez et al. (2006); Puelles et al. (2007); Abellan et al. (2014)	Atoji and Wild, 2004; Atoji and Wild, 2006; Herold et al. (2014)
V	Hp	DGP	Vl
			Tr
DM	APH	APHm, APHi	DM
APHre		APHre	Pa
—		—	Po
—		—	Ma
DL		APHl	DL

APH, area parahippocampalis; APHi, intermediate APH; APHl, lateral APH; APHm, medial APH; APHre, ectopic part of rostral APH; DGP, dentate gyrus primordium; DL, dorsal lateral region of HF; DM, dorsal medial region of HF; Hp, hippocampus, Ma, magnocellular region of HF; Pa, parvocellular region of HF; Po, cell-poor region of HF; Tr, triangular region of HF; V, V-shaped complex; Vl, V-shaped layer region of HF.

## Results

### Selection of Chick Orthologues of Mammalian 5-HTR Genes and LEF1 as APHre Marker Gene

We initially selected the chick orthologs of mammalian *5-HTR* genes, namely *5-HTR1B, 5-HTR1D, 5-HTR1E, 5-HTR1F, 5-HTR2B, 5-HTR3A, 5-HTR5A*, and *5-HTR7*. Among these, the expression patterns of *5-HTR1B* and *5-HTR3A* in the chick telencephalon have been previously reported (Fujita, et al., 2020). The chicken genome also contains *5-HTR6* (International Chicken Genome Sequencing Consortium, 2004); yet we could not obtain a subclone in our study. LEF1 is an important transcription factor for granule cell production in the dentate gyrus (Galceran, et al., 2000). We found that the chick *LEF1* ortholog exhibited sequence similarities with that of humans: 95% (protein) and 85.2% (DNA). In our previous studies, we searched for sequence similarities between chick *5-HTR1B, 5-HTR1D, 5-HTR1E, 5-HTR1F, 5- HTR2B, 5-HTR3A*, *5-HTR5A*, and *5-HTR7* with those from other animals (Fujita et al., 2020; Fujita et al., 2022). The accession numbers and molecular characteristics of ortholog gene probes are summarized in Supplementary Table S2. We accordingly designed probes to detect multiple transcript variants of orthologs registered in the database. Consecutively, we performed *in situ* hybridization (ISH) and analyzed the expression patterns of these orthologs in the telencephalon of chicks.

### Expression of *5-HTR1D* in Chick Telencephalon

**FIGURE 2:**
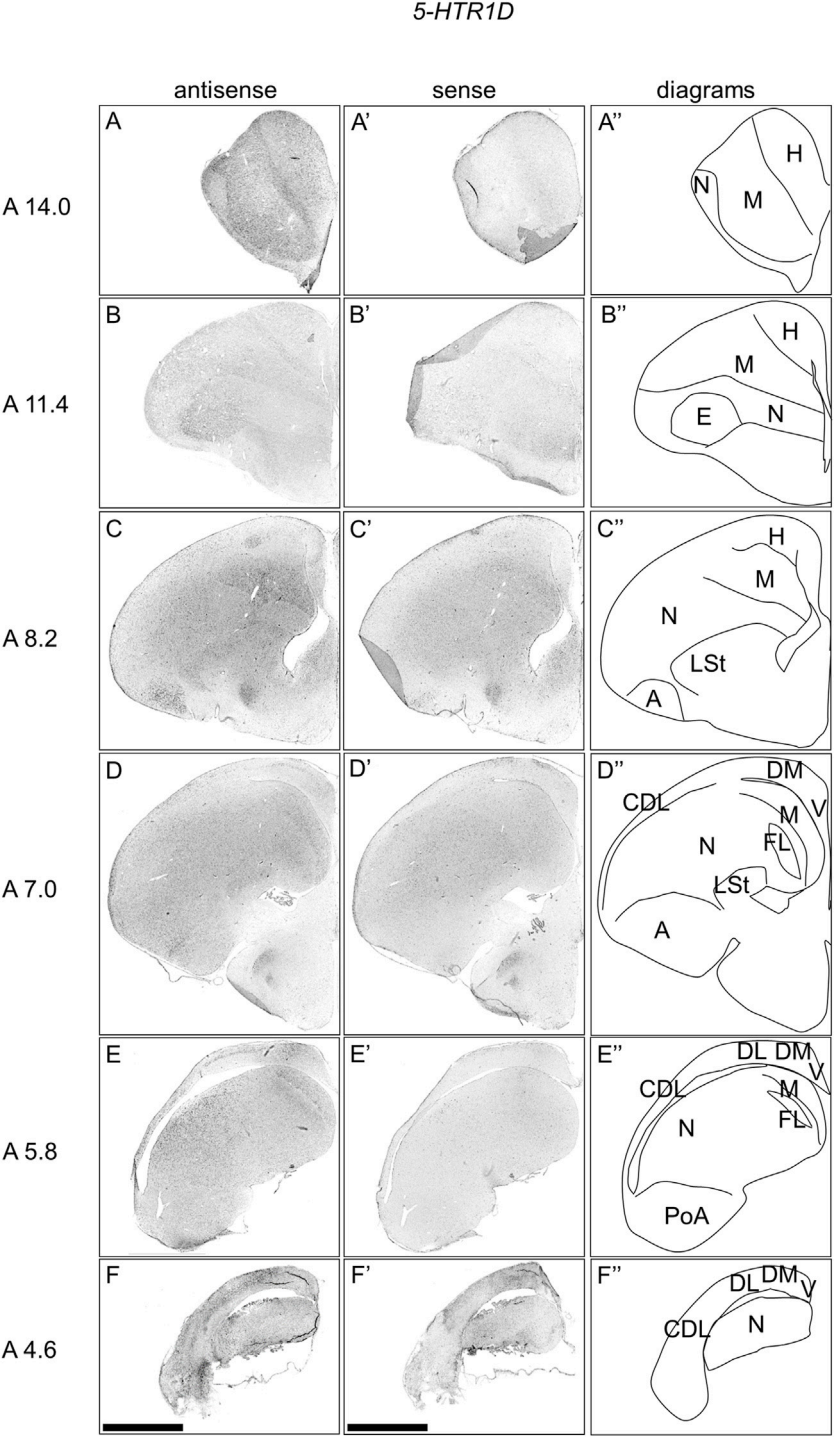
*In situ* hybridization of *5-HTR1D* in P1 chick telencephalons. Digoxigenin-labelled RNA antisense **(A–F)** and sense **(A′–F′)** *5-HTR1D* probes were used for *in situ* hybridization in coronal sections of the P1 chick telencephalon. To evaluate the expression patterns of *5-HTR1D*, sections from seven chicks were analyzed, and representative images from four chick brain sections are shown. Diagrams of coronal sections are shown in the rightmost panels **(A–F″)**. Levels of sections (A 14.0 to A 4.6) were in accordance with those mentioned in Kuenzel and Masson’s chick atlas (Kuenzel and Masson, 1988). A, arcopallium; CDL, area corticoidea dorsolateralis; DL, dorsal lateral region of HF; DM, dorsal medial region of HF; E, entopallium; FL, field L; H, hyperpallium; LSt, lateral striatum; M, mesopallium; N, nidopallium; PoA, posterior pallial amygdala; V, V-shaped complex; P1: posthatch day 1. Scale bars = 2.5 mm.

To comprehensively examine the expression pattern of *5-HTR1D* in the chick telencephalon, we performed ISH using the *5-HTR1D* probe on coronal sections at approximately A14.4 to A 4.6 of naive chicks on posthatch day 1 (P1). We detected signals in a large part of the mesopallium (Figure 2A–E, A′-E′), entopallium (Figure 2B, B′), field L (Figures 2D,E, D′-E′), a part of the hyperpallium (Figure 2C, C′), a large part of the arcopallium (Figure 2C,D, C′-D′), and the lateral part of the nidopallium (Figure 2C–F, C′-F′). In addition, we detected signals in a part of the DM (Figure 2C–F, C′-F′), DL in a characteristic layered manner (Figure 2C–F, C′-F′), and the corticoidea dorsolateralis area (CDL) (Figure 2D–F, D′-F′).

### Expression of *5-HTR1E* in Chick Telencephalon

**FIGURE 3:**
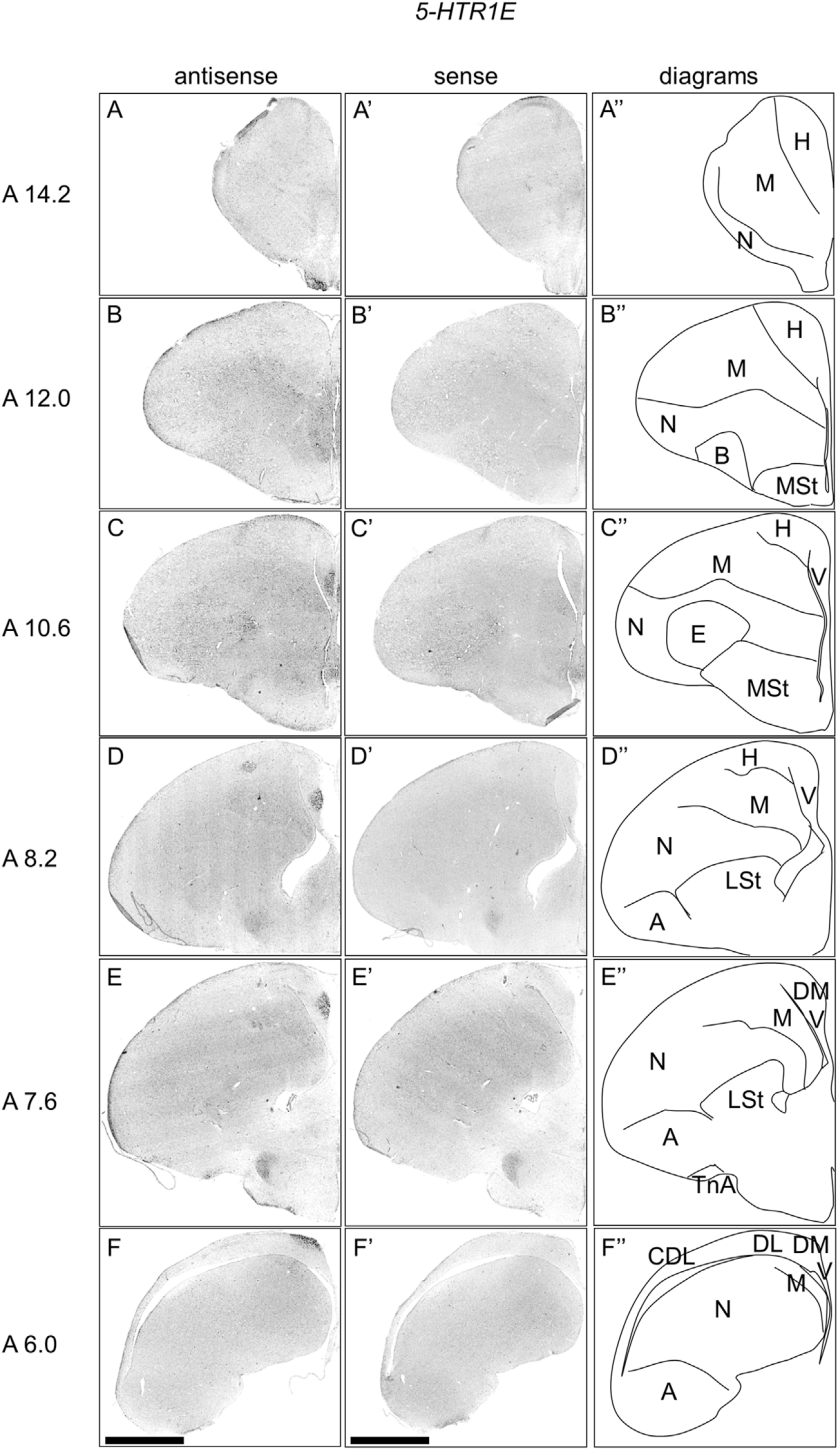
*In situ* hybridization of *5-HTR1E* in P1 chick telencephalons. Digoxigenin-labelled RNA antisense **(A–F)** and sense **(A′–F′)** *5-HTR1E* probes were used for *in situ* hybridization in coronal sections of the P1 chick telencephalon. To evaluate the expression patterns of *5-HTR1E*, sections from nine chicks were analyzed, and representative images from three chick brain sections are shown. Diagrams of coronal sections are shown in the rightmost panels **(A–F″)**. Levels of sections (A 14.2 to A 6.0) were in accordance with those mentioned in Kuenzel and Masson’s chick atlas (Kuenzel and Masson, 1988). A: arcopallium; B: basorostralis; CDL: the area corticoidea dorsolateralis; DL: the dorsal lateral region of HF; DM: the dorsal medial region of HF; E: entopallium; H: hyperpallium; LSt lateral striatum; M, mesopallium; MSt, medial striatum; N, nidopallium; TnA, nucleus taeniae of the amygdala; V, V-shaped complex; P1: posthatch day 1. Scale bars = 2.5 mm.

We examined the expression pattern of 5-*HTR1E* in sections A14.2 to A6.0 of P1 chick telencephalons (Figure 3). We detected strong signals in the nucleus taeniae of the amygdala (TnA) (Figure 3E, E′), and a part of the DM, in a cluster manner (Figure 3C–F, C′-F′). We also detected signals in a part of the mesopallium (Figure 3A–F, A′-F′), hyperpallium (Figure 3D, D′), DL in a layered manner (Figure 3D–F, D′-F′), and CDL (Figure 3F, F′).

### Comparison of *5-HTR1E* and *LEF1* Expression Patterns in Chick APH

**FIGURE 4:**
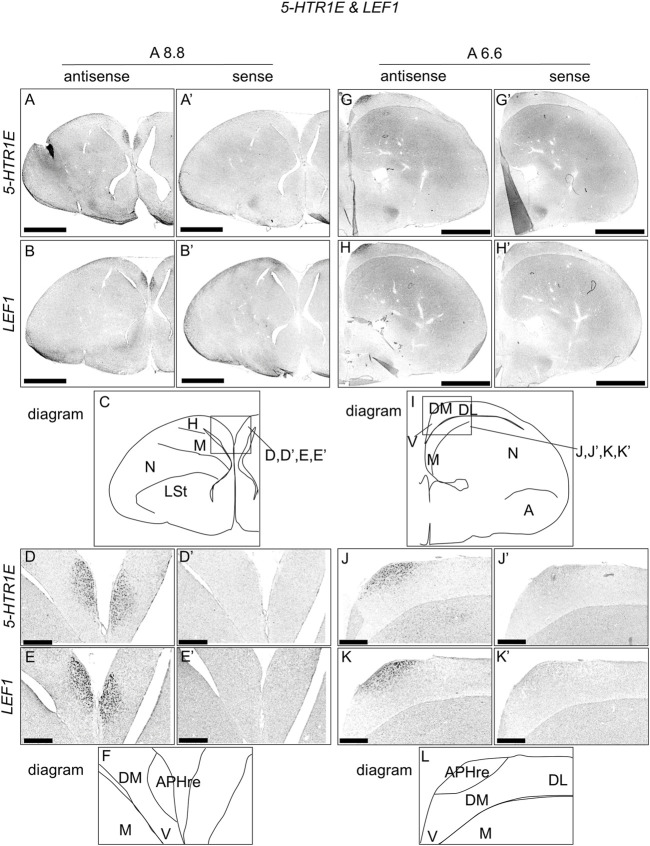
Comparison of *5-HTR1E* and *LEF1* expression patterns in P1 chick telencephalon sections. *In situ* hybridization using DIG-labelled RNA antisense and sense *5-HTR1E* **(A,D,G,J**, **and** **A′,D′,G′,J′)**, respectively, and *LEF1* **(B,E,H,K**, **and** **B′,E′,H′,K′)**, respectively probes in P1 chick brain coronal sections are shown. Panels **(C) (F) (I),** and **(L)** show diagrams of **(A) (D) (G),** and **(J),** respectively. Levels of sections (A8.8 and A6.6) were in accordance with those mentioned in Kuenzel and Masson’s chick atlas (Kuenzel and Masson, 1988). A: arcopallium; APHre: ectopic part of the rostral area hippocampalis; DL, dorsal lateral region of HF; DM, dorsal medial region of HF; H, hyperpallium; LSt, lateral striatum; M, mesopallium; N, nidopallium; V, V-shaped complex; P1: posthatch day 1. Scale bars = 2.5 mm **(A,B,G,H,A′,B′,G′,H′)** and 500 µm **(D,E,J,K,D′,E′,J′,K′)**.

To compare the expression patterns between *5-HTR1E* and *LEF1*, which is the APHre region marker (Abellan et al., 2014), we performed ISH using *5-HTR1E* and *LEF1* probes in neighboring sections of A8.8 and A6.6, respectively (Figure 4). Interestingly, we found that the regions of expression of *5-HTR1E* and *LEF1* in the APHre matched well at both sections (Figure 4D,E, J-K, D′-E′, J′-K′).

### Expression of *5-HTR1F* in Chick Telencephalon

**FIGURE 5:**
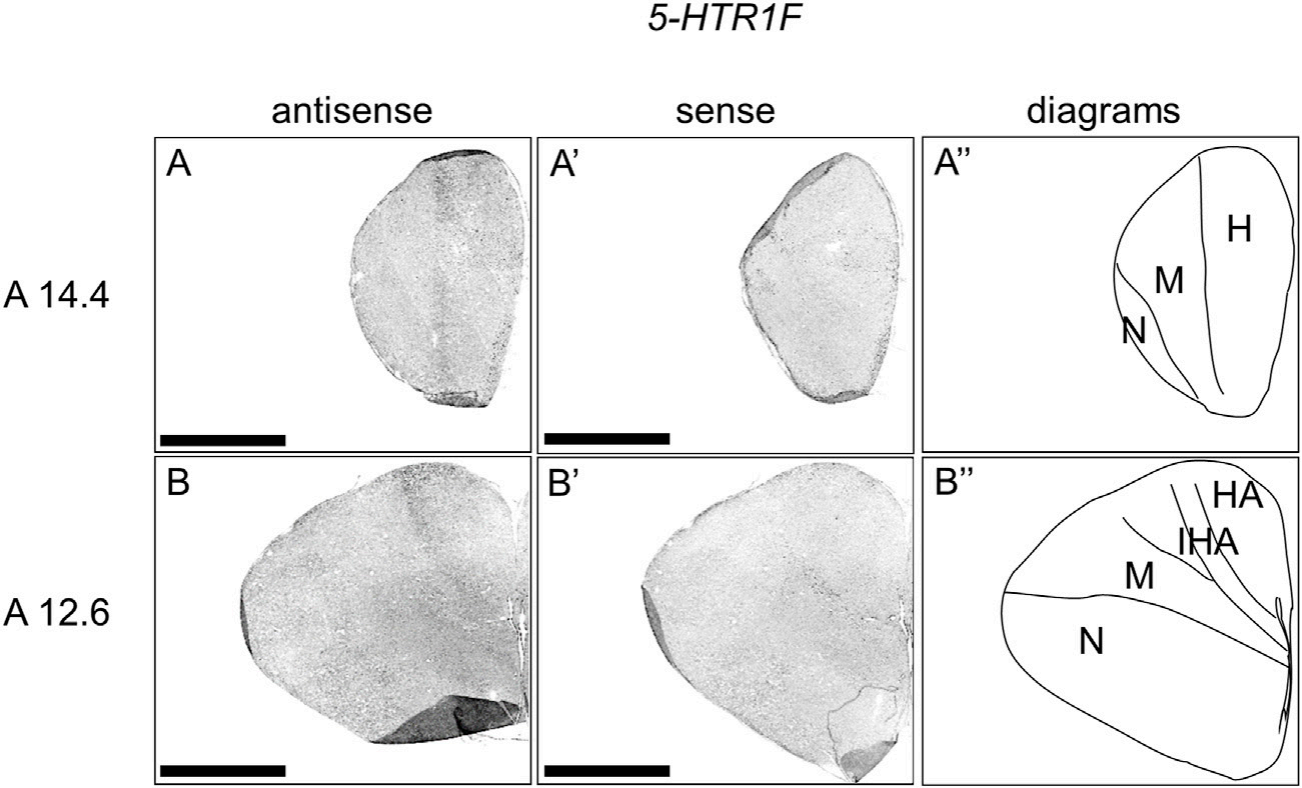
*In situ* hybridization of *5-HTR1F* in P1 chick telencephalon. Digoxigenin-labelled RNA antisense **(A–B)** and sense **(A′–B′)** *5-HTR1F* probes were used for *in situ* hybridization in coronal sections of the P1 chick telencephalon. To evaluate the expression patterns of *5-HTR1F*, sections from eight chicks were analyzed, and representative images from two chick brain sections are shown **(A″–B″)** Diagrams of coronal sections are shown in the rightmost panels. Levels of sections (A14.4 and A12.6) were in accordance with those mentioned in Kuenzel and Masson’s chick atlas (Kuenzel and Masson, 1988). H, hyperpallium; HA, hyperpallium apicale; IHA, interstitial part of the hyperpallium; M, mesopallium; N, nidopallium; P1: posthatch day 1. Scale bars = 2.5 mm.

We then examined the expression patterns of *5-HTR1F* in sections A14.4 to A5.0 of P1 chick telencephalons (Figure 5). Our analysis revealed the presence of signals in the interstitial part of the hyperpallium (Figure 5A,B, A′-B′).

### Expression of *5-HTR5A* in Chick Telencephalon

**FIGURE 6:**
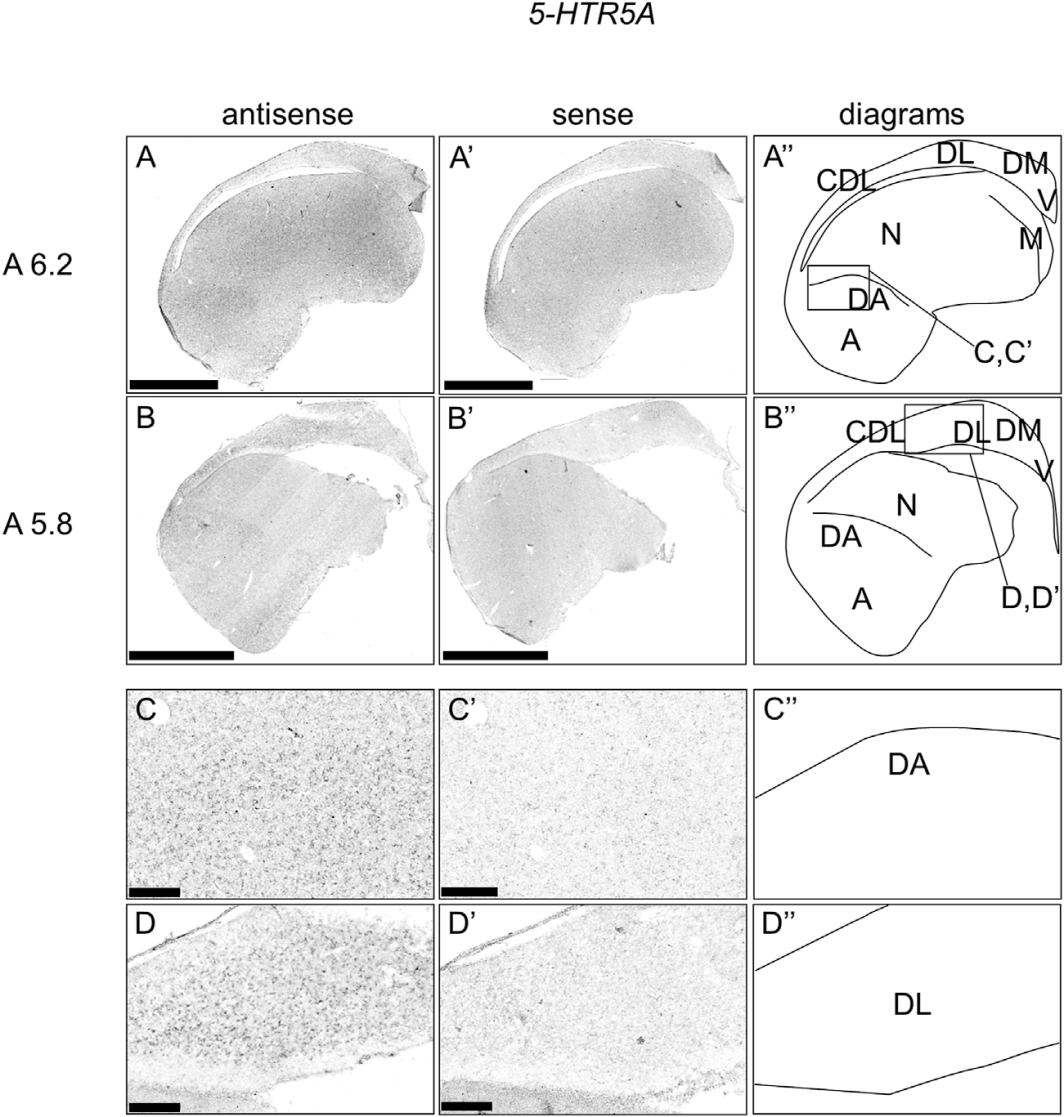
*In situ* hybridization of *5-HTR5A* in P1 chick telencephalon. Digoxigenin-labelled RNA antisense **(A–D)** and sense **(A′–D′)** *5-HTR5A* probes were used for *in situ* hybridization in coronal sections of the P1 chick telencephalon. To evaluate the expression patterns of *5-HTR5A*, sections from six chicks were analyzed, and representative images from two chick brain sections are shown **(A″–D″)** Diagrams of coronal sections are shown in the rightmost panels. Levels of sections (A 6.2 and A 5.8) are in accordance with those mentioned in Kuenzel and Masson’s chick atlas (Kuenzel and Masson, 1988) **(C–D** **and** **C′–D′)** Magnified views of brain areas shown in the boxes in (A″ and B″). A: arcopallium; CDL, area corticoidea dorsolateralis; DA, dorsal arcopallium; DL, dorsal lateral region of HF; DM, dorsal medial region of HF; M, mesopallium; N, nidopallium; V, V-shaped complex; P1: posthatch day 1. Scale bars = 2.5 mm **(A–B** **and** **A′–B′)** and 250 µm **(C–D** **and** **C′–D′)**.

We also examined the expression patterns of 5-*HTR5A* in sections A13.8 to A4.4 of P1 chick telencephalons (Figure 6). We accordingly detected the expression of 5-*HTR5A* in the dorsal arcopallium, lateral nidopallium, DL, and CDL (Figure 6A,B, A′-B′).

### Expression of *5-HTR7* in Chick Telencephalon

**FIGURE 7:**
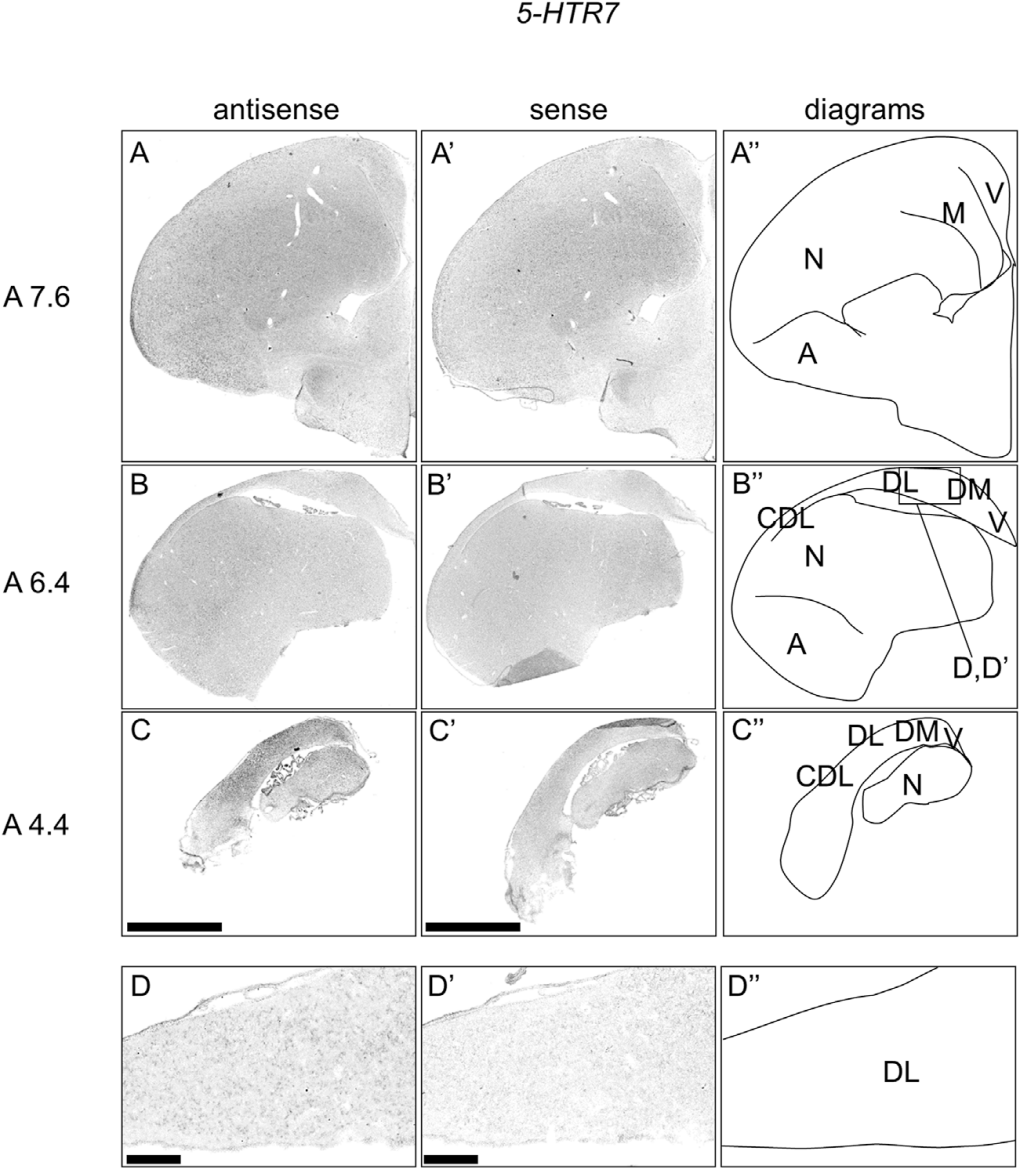
*In situ* hybridization of *5-HTR7* in P1 chick telencephalons. Digoxigenin-labelled RNA antisense **(A–D)** and sense **(A′–D′)** *5-HTR7* probes were used for *in situ* hybridization in coronal sections of the P1 chick telencephalon. To evaluate the expression patterns of *5-HTR7*, sections from six chicks were analyzed, and representative images from three chick brain sections are shown **(A″–D″)** Diagrams of coronal sections are shown in the rightmost panels. Levels of sections (A 7.6, A 6.4, and A 4.4) were in accordance with those mentioned in Kuenzel and Masson’s chick atlas (Kuenzel and Masson, 1988) **(D** **and** **D′)** Magnified views of brain areas shown in the boxes in **(B″)**. A, arcopallium; CDL, area corticoidea dorsolateralis; DL, dorsal lateral region of HF; DM, dorsal medial region of HF; M, mesopallium; N, nidopallium; V, V-shaped complex; P1: posthatch day 1. Scale bars = 2.5 mm **(A–C** **and** **A′–C′)** and 250 µm **(D** **and** **D′)**.

We further examined the expression patterns of *5-HTR7* in sections A13.6 to A4.4 of P1 chick telencephalons (Figure 7) and found that *5-HTR7* was mainly expressed in a large part of the arcopallium, lateral nidopallium, DM, DL (Figure 7A–C, A′-C′), and CDL (Figure 7B,C, B′-C′).

### Expression of *5-HTR2B* in Chick Telencephalon

Finally, we examined the expression patterns of *5-HTR2B* in sections A13.2 to A5.0, but did not detect any signal (data not shown), suggesting that the levels of expression of *5-HTR2B* were either very low or cells expressing *5-HTR2B* were very rare.

### Comparison of *5-HTR1B, 5-HTR1D, 5-HTR1E,* and *5-HTR3A* Expression Patterns in Chick Hippocampal Formation

**FIGURE 8:**
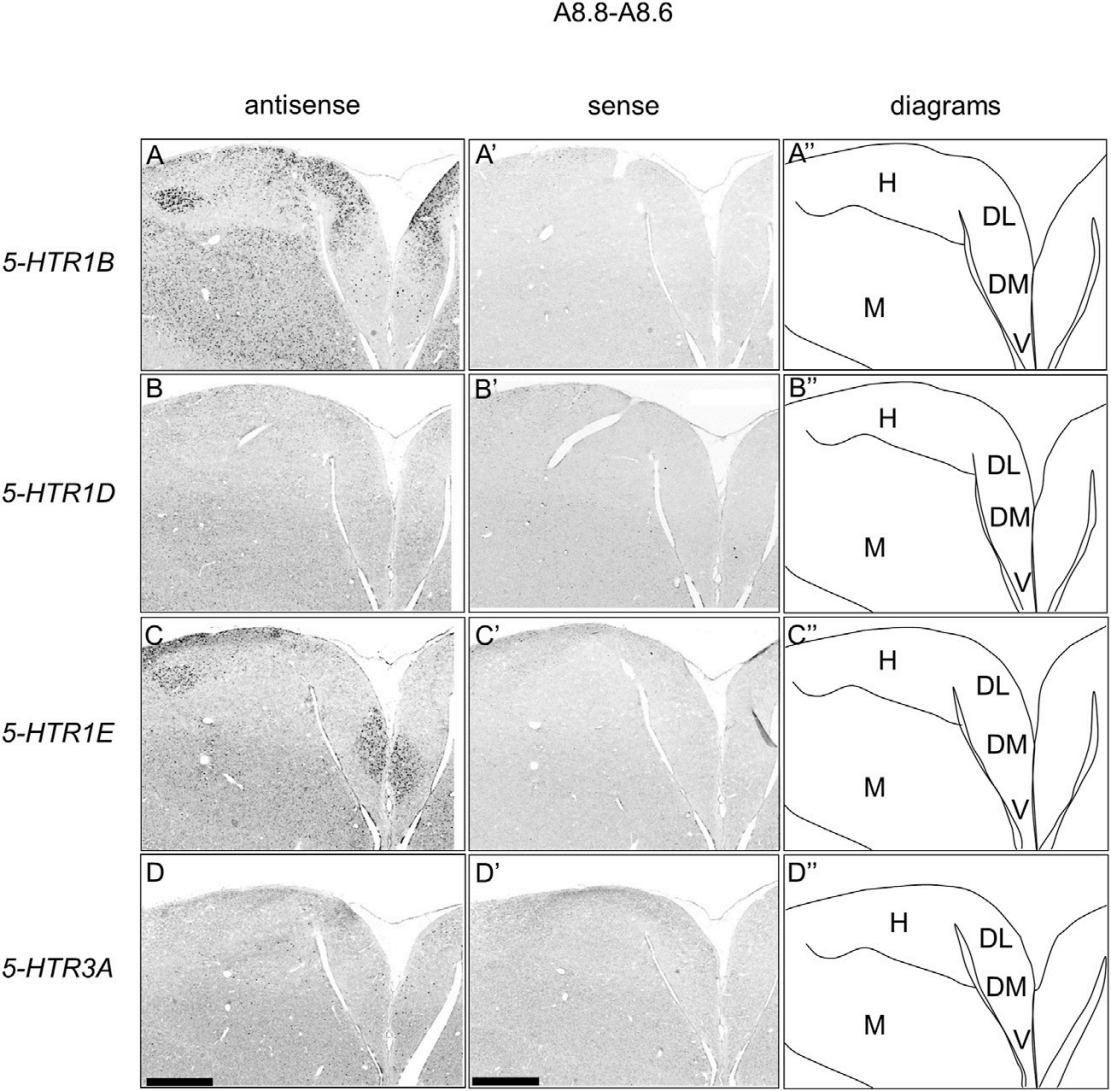
Comparison of *5-HTR*s expression patterns in HF in P1 chick telencephalons using neighboring sections at approximately A 8.8 level. *In situ* hybridization using DIG-labelled RNA antisense and sense *5-HTR1B* **(A** **and** **A′)**, *5-HTR1D* **(B and B′)**, *5-HTR1E* **(C** **and** **C′)**, and *5-HTR3A* **(D** **and** **D′)** probes in coronal sections of P1 chick telencephalons **(A″–D″)** Diagrams of coronal sections are shown in the rightmost panels. DL, dorsal lateral region of HF; DM, dorsal medial region of HF; H, hyperpallium; M, mesopallium; V, V-shaped complex; P1: posthatch day 1. Scale bars = 1 mm.

**FIGURE 9:**
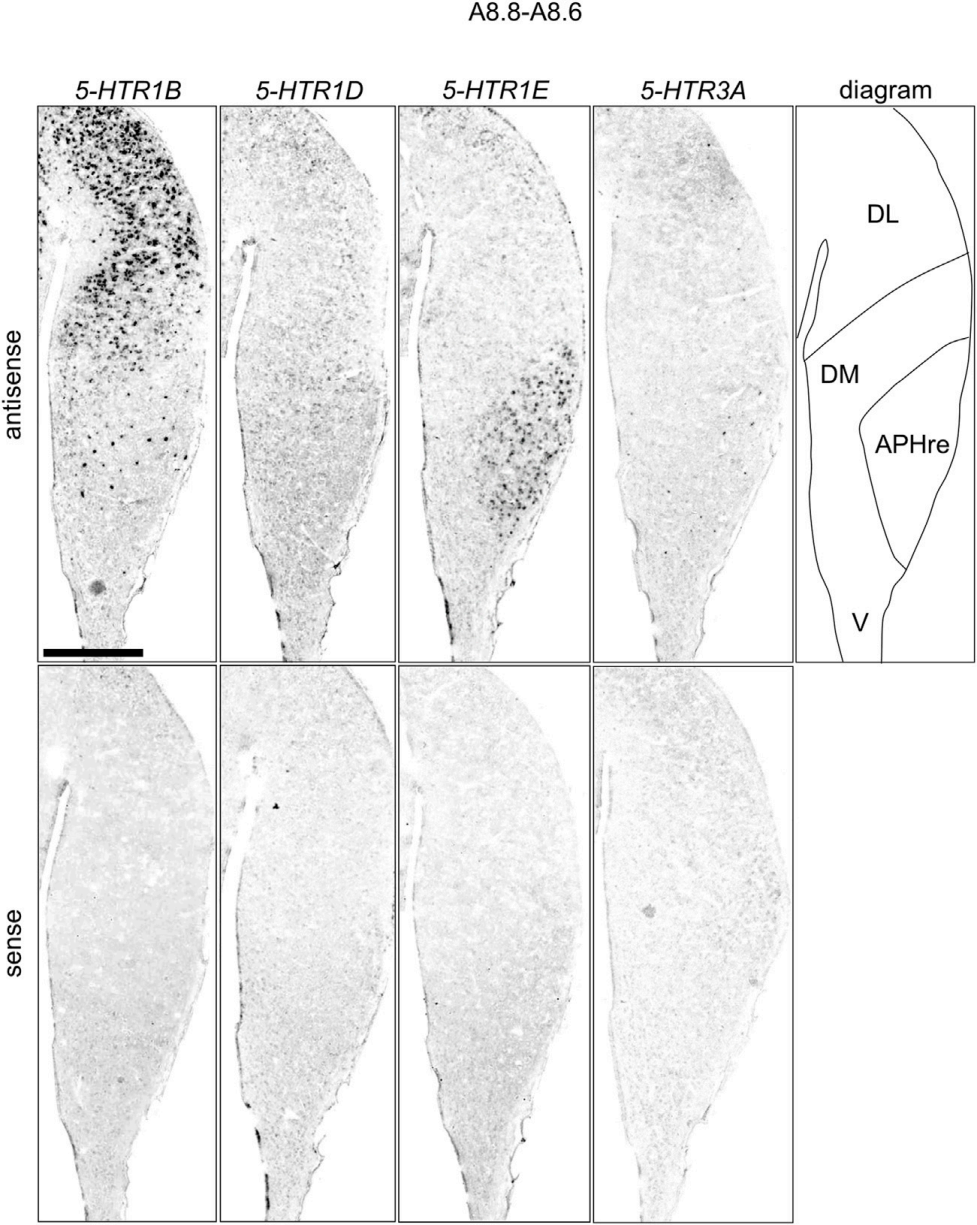
Comparison of *5-HTR*s expression patterns in HF in P1 chick telencephalons using neighboring sections at approximately A 8.8 level focused on HF. Magnified views of HF surrounding regions are shown in Figure 8. APHre: ectopic part of the rostral area hippocampalis; DL: dorsal lateral region of HF; DM: dorsal medial region of HF; V: V-shaped complex; P1: posthatch day 1. Scale bars = 1 mm.

**FIGURE 10:**
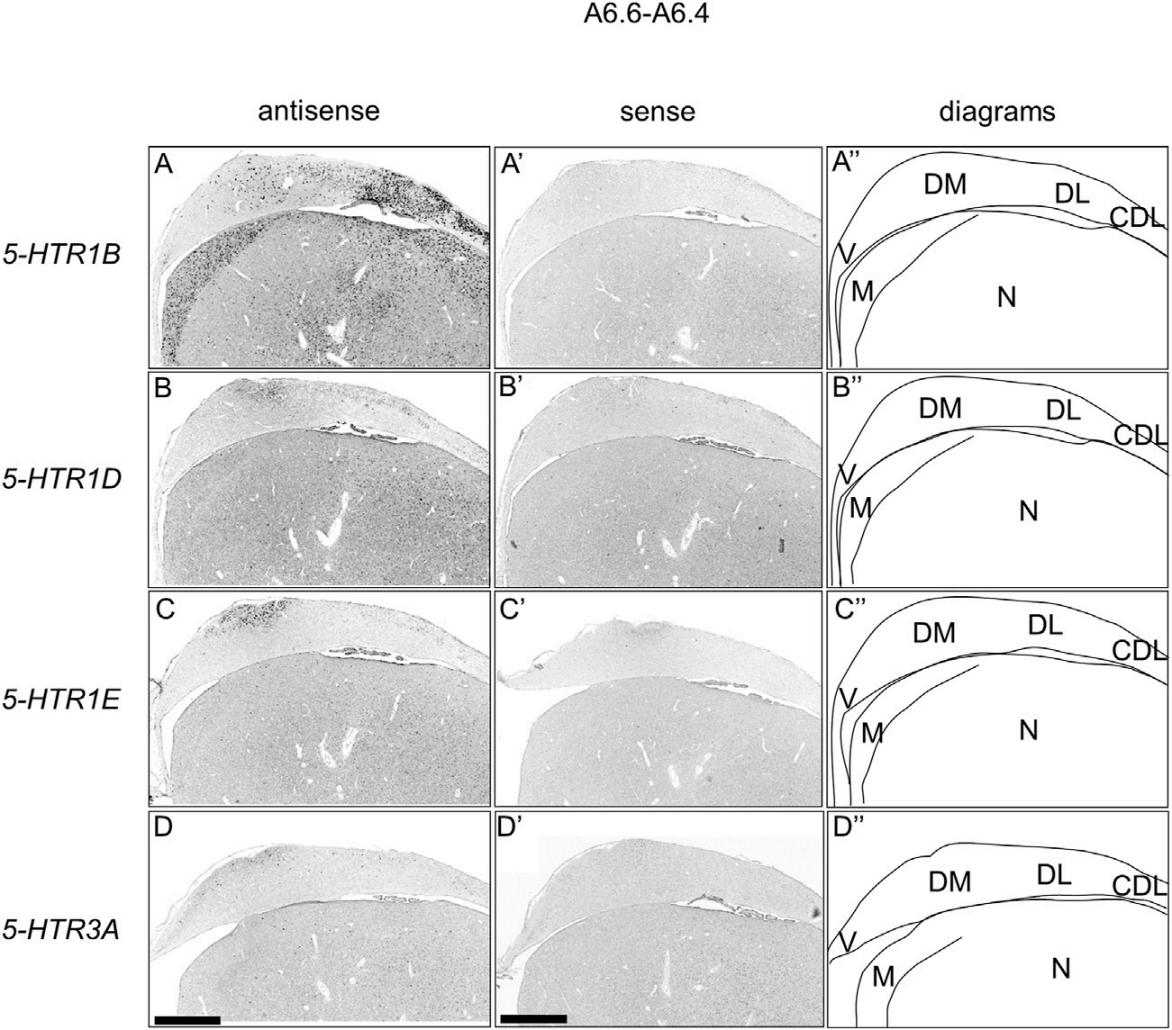
Comparison of *5-HTR*s expression patterns in HF in P1 chick telencephalons using neighboring sections at approximately A 6.6. *In situ* hybridization using DIG-labelled RNA antisense and sense *5-HTR1B* **(A and A′)**, *5-HTR1D* (B and B′), *5-HTR1E* **(C** **and** **C′)**, and *5-HTR3A* **(D** **and** **D′)** probes in coronal sections of P1 chick telencephalons **(A″–D″)** Diagrams of coronal sections are shown in the rightmost panels. CDL: area corticoidea dorsolateralis; DL: dorsal lateral region of HF; DM: dorsal medial region of HF; M: mesopallium; N: nidopallium; V: V-shaped complex; P1: posthatch day 1. Scale bars = 1 mm.

**FIGURE 11:**
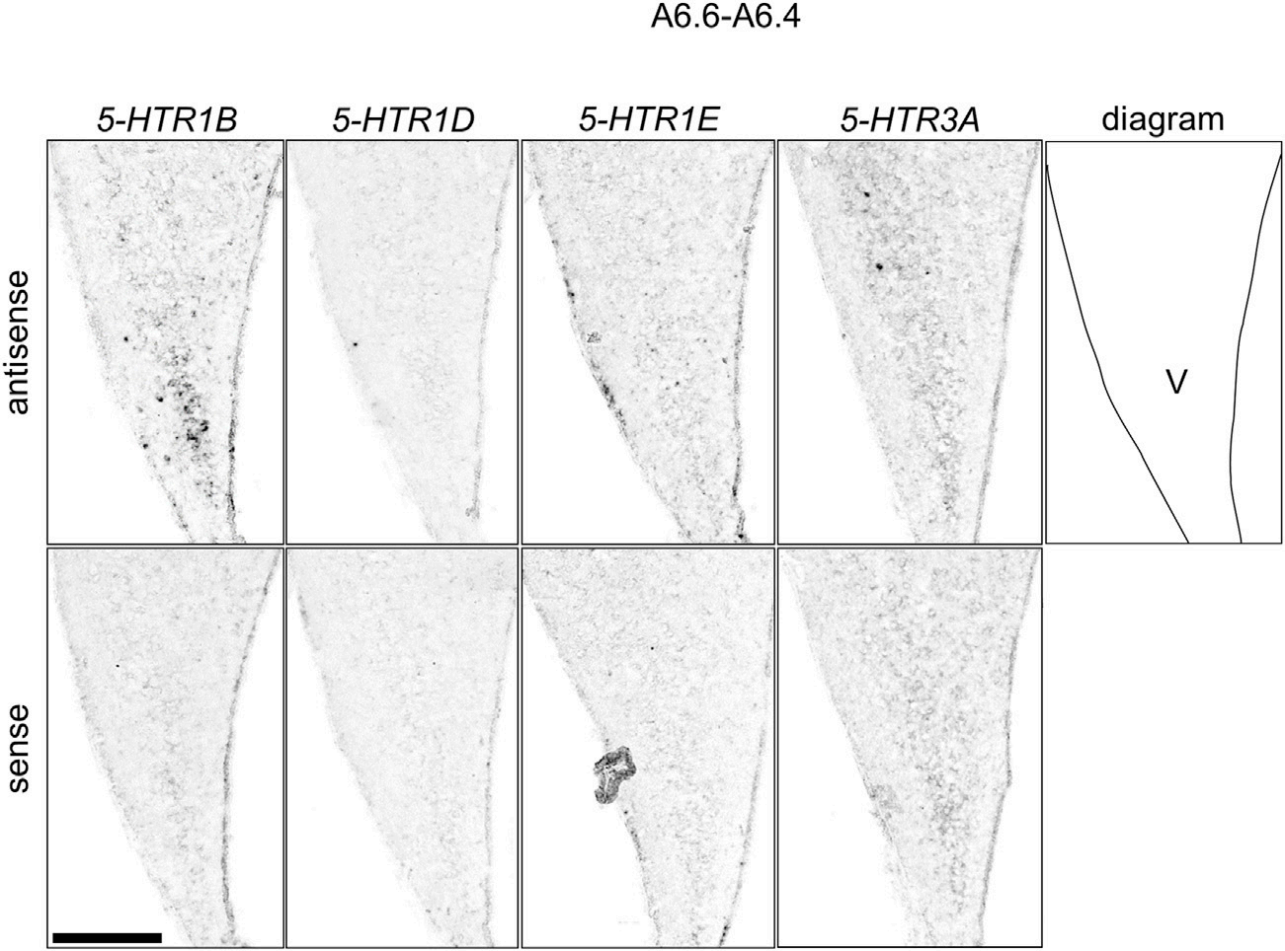
Comparison of *5-HTR*s expression patterns in the HF in the P1 chick telencephalons using neighboring sections around A 6.6 focused on V. Magnified views of HF surrounding regions shown in Figure 10. V: V-shaped complex; P1: posthatch day 1. Scale bars = 250 µm.

**FIGURE 12:**
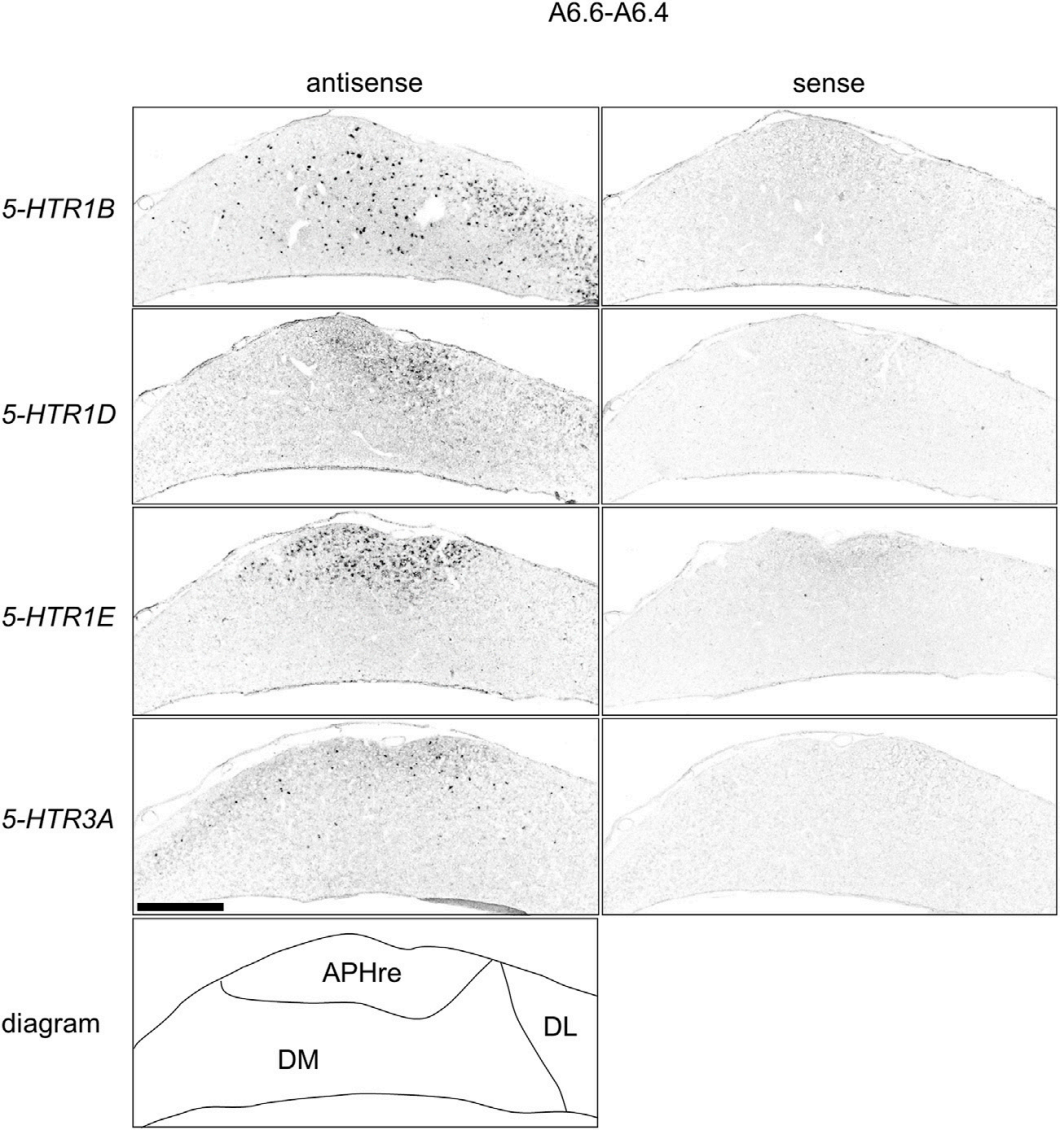
Comparison of *5-HTR*s expression patterns in HF in P1 chick telencephalons using neighboring sections at approximately A 6.6 focused on DM. Magnified views of HF surrounding regions shown in Figure 10. APHre, ectopic part of the rostral area parahippocampalis; DL, dorsal lateral region of HF; DM, dorsal medial region of HF; P1: posthatch day 1. Scale bars = 500 µm.

**FIGURE 13:**
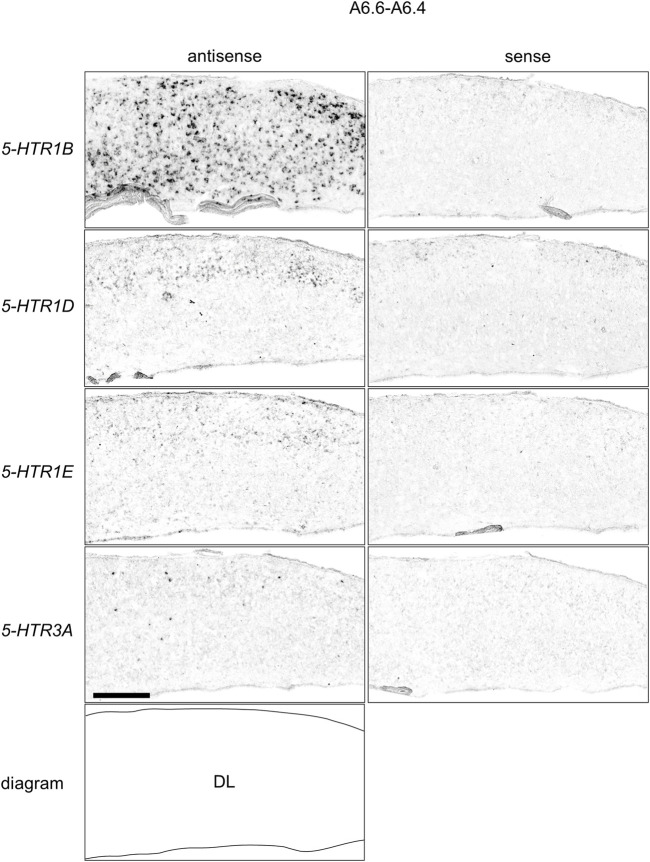
Comparison of *5-HTR*s expression patterns in HF in P1 chick telencephalons using neighboring sections at approximately A 6.6 focused on DL. Magnified views of HF surrounding regions shown in Figure 10. DL: dorsal lateral region of HF; P1: posthatch day 1. Scale bars = 250 µm.

We found that *5-HTR1D* and *5-HTR1E* were clearly expressed in chick HF. In our previous study, we revealed the characteristic expression patterns of *5-HTR1B* and *5-HTR3A* (Fujita, et al., 2020). Subsequently, to understand the relationship between the regions of expression of these *5-HTR*s in chick HF, we performed ISH using *5-HTR1B, 5-HTR1D, 5-HTR1E,* and *5-HTR3A* probes on neighboring sections of A8.8 to A8.6 (Figures 8, 9A6.6 to A6.4 (Figures 10–13). We found that *5-HTR1B* was expressed in the whole DL (Figure 8A, A′, Figure 9, Figure 10A, A′, Figure 13), whereas sparsely in the DM (Figure 8A, A′, Figure 9, Figure 10A, A′, Figure 12). We also detected the expression of *5-HTR1D* in a part of DM (Figure 8B, B′, Figure 9, Figure 10B, B′, Figure 12), and in DL in a layered manner (Figure 8B, B′, Figure 9, Figure 10B, B′, Figure 13). We found that *5-HTR1E* was expressed in APHre (Figure 8C, C′, Figure 9, Figure 10C, C′, Figure 12), and in DL in a layered manner (Figure 8C, C′, Figure 9, Figure 10C, C′, Figure 13). We detected sparse signals of expression of *5-HTR3A* in both the DM and DL (Figure 8D, D′, Figure 9, Figure 10D, D’, Figure 12, Figure 13). Finally, we observed that *5-HTR1B* and *5-HTR3A* were sparsely expressed in V, whereas we detected a faint expression of 5-*HTR1E* and no expression of *5-HTR1D* in this region (Figure 11).

## Discussion

### Expression Patterns of *5-HTR* Subfamily Genes in Chick HF

**FIGURE 14:**
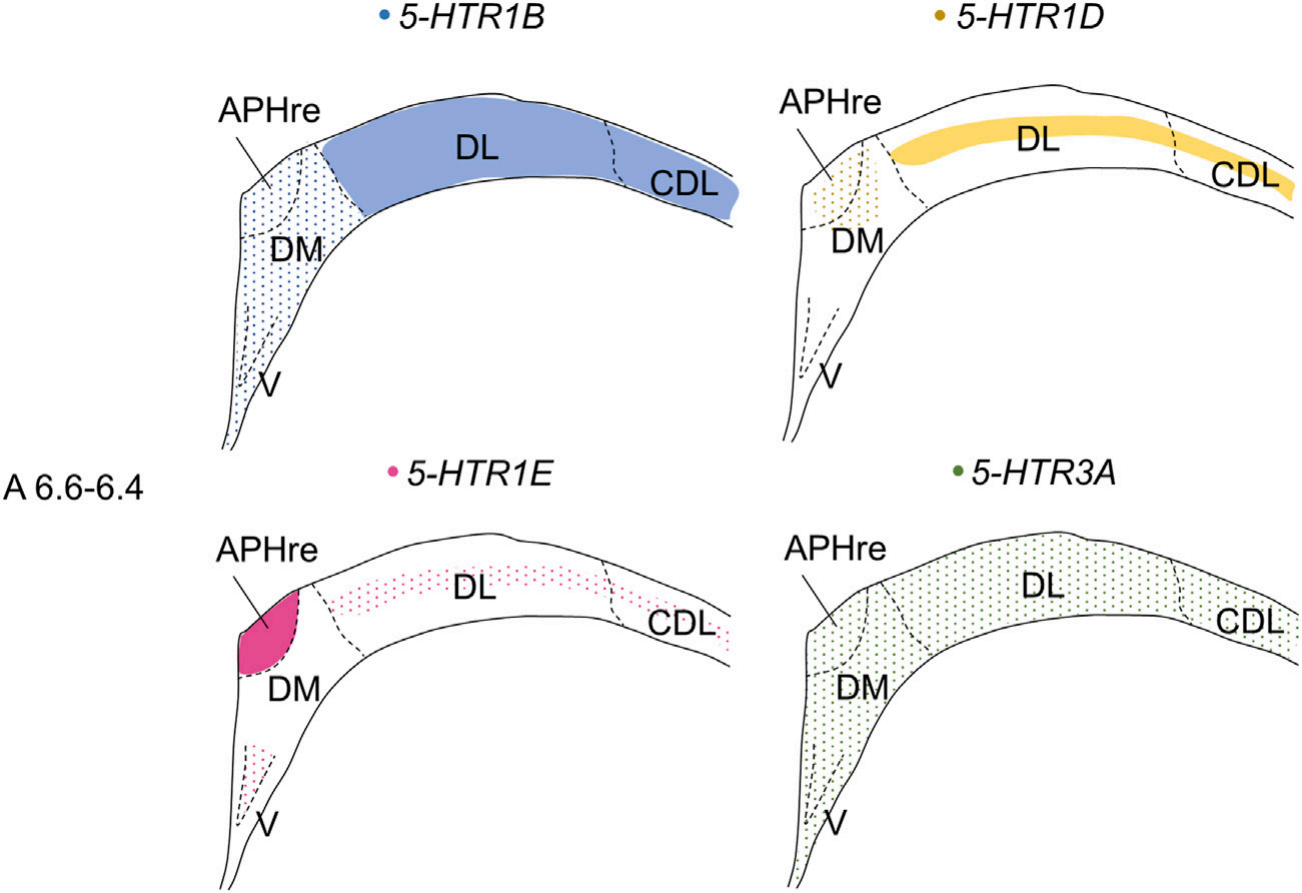
Schematic summary of expression patterns of *5-HTR1B, 5-HTR1D, 5-HTR1E,* and *5-HTR3A* in subdivisions in the P1 chick HF. Representative expression patterns in sections at approximately A 6.6 to A 6.4 are shown in colored areas (blue, *5-HTR1B*; yellow, *5-HTR1D*; magenta, *5-HTR1E*; green, *5-HTR3A*). A dotted pattern indicates the sparse distribution of expressing cells. Levels of sections were in accordance with those mentioned in Kuenzel and Masson’s chick atlas (Kuenzel and Masson, 1988). APHre: ectopic part of the rostral area parahippocampalis; CDL, corticoidea dorsolateralis; DL, dorsal lateral region of HF; DM, dorsal medial region of HF; V, V-shaped complex; P1: posthatch day 1.

In the present study, we revealed that 4 *5-HTR*s, *5-HTR1B, 5-HTR1D, 5-HTR1E*, and *5-HTR3A*, were expressed in a subdivision- and layer-selective manner in chick HF (Figure 14). We also found that *5-HTR5A* and *5-HTR7,* which were faintly expressed, did not clearly show subdivision- or layer-selective expression patterns in the chick HF.

We found that *5-HTR1B* was highly expressed in the DL, whereas sparsely in V, DM, APHre, while *5-HTR3A* was expressed in all the V, DM, DL, APHre, sparsely. In comparison, in mammals, *5-Htr1b* is expressed in Cornu Ammonis 1 (CA1) and CA3 pyramidal cells, GABAergic interneurons of the hilus, granule cells of dentate gyrus (DG), the subgranular zone, the layer II pyramidal cells in the entorhinal cortex (EC) (Tanaka, et al., 2012), while *5-Htr3A* is expressed in GABAergic interneurons of the hilus, in the subgranular zone, in scattered EC cells, CA1, CA3, and DG (Tecott, et al., 1993; Fonseca et al., 2001; Morales and Wang 2002; Tanaka, et al., 2012; Koyama, et al., 2017). Considering the expression patterns of *5-HTR1B* and *5-HTR3A*, our results support the correspondence of V and DM in the avian HF to the mammalian hippocampal proper (DG and CA subfields) (Montagnese et al., 1996; Szekely, 1999; Atoji and Wild, 2004; Atoji and Wild, 2006; Suarez, et al., 2006; Puelles et al., 2007; Herold et al., 2014; Abellan et al., 2014; Atoji et al., 2016; Medina et al., 2017b). To date, a one-to-one correspondence of subdivisions between the avian and mammalian HF has not been established, while various studies have postulated on possible areas in avian HF corresponding to mammalian DG. One suggestion is that the mammalian DG corresponds to V in avian HF (Atoji and Wild, 2004; Suarez, et al., 2006; Puelles et al., 2007; Gupta, et al., 2012; Herold et al., 2014; Atoji et al., 2016). Another theory is that the mammalian DG corresponds to DM in avian HF (Montagnese et al., 1996; Szekely, 1999). However, another possibility is that DG might be a novel acquisition in mammals, which would imply that there is no homolog in birds. The avian HF has undergone divergence over hundreds of millions of years of evolution, thus making it difficult to compare the subdivisions of HF between birds and mammals (Hevner, 2016; Striedter, 2016). In fact, although no macrostructure corresponding to mossy fibers in the mammalian DG has been observed in avian HF (Faber et al., 1989; Montagnese et al., 1993, 1996; Tombol et al., 2000; Herold, et al., 2014), this does not exclude the existence of a neuron population in the avian HF corresponding to granule cells in the mammalian DG. This candidate neuronal cell population corresponding to granule cells might be unevenly distributed in the avian HF. We assumed that using chick orthologs of another marker gene to distinguish pyramidal from granule cells in mammalian HF could provide new clues for the correspondence between avian and mammalian HF.

In chicks, *5-HTR1E* was highly expressed in APHre, and weakly in the V and DL in a layer-selective manner, suggesting that the APHre cell population that preferentially expresses *5-HTR1E* has novel characteristics. In contrast, in mammals, *5-Htr1e* was shown to be expressed in the CA fields and DG (Bruinvels et al., 1994a; Mengod, et al., 2006; Vilaro et al., 2020). However, because the relationship between APHre in avian HF and other HF subdivisions and the function of APHre are completely unknown (Abellan et al., 2014), it was difficult to determine the correspondence of APHre to the mammalian subdivision of HF. In the future, a better understanding of the features of APHre in chicks with respect to multiple aspects, such as connectivity, electrophysiological properties, and behavioral function, is expected to help determine the correspondence of APHre subdivisions.

Patch RNA-sequencing analysis in mammals showed that *5-Htr1d* was expressed in the GABAergic interneurons of CA1 (Luo, et al., 2019). We here found that *5-HTR1D* was expressed in DM, APHre, and DL in a layer-selective manner. Based on this, we assumed that *5-HTR1D* might also be expressed in GABAergic interneurons in chick HF.

In the past, the cytoarchitecture of avian HF was considered quite different from that of the mammalian 3-layered HF, as it appeared to have completely lost its layered structure. Three layers are clearly present in lizards but less visible in crocodiles (closest to birds) as for HF homologue. (Hevner, 2016; Striedter, 2016). However, detailed immunohistochemical analysis and combinatorial expression analysis using developmental regulatory genes revealed the existence of a layered cytoarchitecture orthogonal to radial glial fibers in the chick HF during its developmental stages (Redies et al., 2001; Abellan et al., 2014). Such a layered structure has also been confirmed in the HF of adult pigeons (Suarez et al., 2006; Herold et al., 2019). In our study, we showed that the expression patterns of *5-HTR1D* and *5-HTR1E* in DL were layered, suggesting a selective regulation of the DL layer by these receptors. Our data indicated that the neuronal population in this layer might have functional roles in cognition and emotion.

We also examined the expression of *5-HTR2B* and failed to detect it in chick telencephalons, suggesting either a low level of expression of *5-HTR2B* or the rarity of expressing cells. Interestingly, the expression level of *5-Htr2b* in the telencephalon of mammals was found to be low (Bonaventure et al., 2002; Vilaro, et al., 2020). This finding was consistent with our obtained results for the expression of *5-HTR2B*.

### Possible Functions of Chick 5-HTRs in Chick Telencephalon Other Than HF

Regarding its the expression pattern, we noticed that *5-HTR1D* was expressed in a large part of the mesopallium, arcopallium, and a part of the hyperpallium, nidopallium, HF, CDL, and major part of the intercalated nidopallium (entopallium and field L (Jarvis et al., 2013)). Of note, the intercalated nidopallium receives sensory projections from the thalamus (Reiner et al., 2004; Jarvis et al., 2013). We previously showed that *5-HTR2C* was preferentially expressed in intercalated nidopallium in chicks (Fujita et al., 2020). Considering the expression combination of *5-HTR1D* and *5-HTR2C*, it is possible that these 2 5-HTRs might work together in sensory input information processing in the intercalated nidopallium in birds. In mammals, 5-HTR1D was demonstrated to be distributed in the frontoparietal cortex, primary olfactory cortex, accumbens nucleus, caudate-putamen, and lateral mammillary nucleus (Bruinvels, et al., 1994a, 1994b; Vilaro et al., 2020). However, the regional expression of *5-HTR1D* in the chick hyperpallium appeared to be limited (Figure 2C, C’), suggesting a serotonergic modulation in the neuronal population of the hyperpallium via 5-HTR1D.

In the case of the expression pattern of *5-HTR1E*, we found that the major regions of expression were the APHre, TnA, and a part of the hyperpallium. We previously showed that *5-HTR2C* and *5-HTR4* were preferentially expressed in the TnA in chicks (Fujita, et al., 2020), whereas in mammals, *5-HTR2C* and *5-HTR4* are expressed in the amygdala (Huang and Kandel, 2007; Bombardi 2014; Bocchio et al., 2016). TnA is considered to be the counterpart of the mammalian medial amygdala (Reiner et al., 2004; Yamamoto et al., 2005; Yamamoto and Reiner, 2005; Hanics et al., 2017), which is functionally associated with social behaviors, including sexual behavior and social interactions (Ikebuchi, et al., 2009; Mayer et al., 2017; Mayer, et al., 2019). These findings indicated that 5-HT might play a key role in shaping social responses in birds and mammals. In this study, we found another *5-HTR*, *5-HTR1E*, which was preferentially expressed in TnA. Whereas, in mammals, 5-*Htr1e* is expressed in the amygdala (Lowther et al., 1992; Bruinvels et al., 1994a; Mengod et al., 2006). Our findings regarding *5-HTR1E* also support the potentially conserved roles of *5-HTR*s in the mammalian medial amygdala and avian TnA. Furthermore, the expression patterns of *5-HTR1E* and *5-HTR1D* in the hyperpallium, appeared to be similar (Figure 8A,C, A′, C’), suggesting a serotonergic modulation in the neuronal population of the hyperpallium via these receptors.

Regarding the expression pattern of *5-HTR1F*, we observed that it was selectively expressed in the interstitial part of the hyperpallium (IHA). According to developmental studies, the hyperpallium of birds is a region homologous to the mammalian neocortex (Fernandez, et al., 1998; Puelles et al., 2000), and is composed of four pseudolayers: the apical part of the hyperpallium, IHA, the intercalated part of the hyperpallium, and the densocellular part of the hyperpallium (Medina and Reiner 2000; Reiner et al., 2004). Among them, the IHA has projection terminals of sensory information from the thalamus, which is considered equivalent to layer IV of the mammalian neocortex (Bangnoli and Burkhalter, 1983; Miceli and Reperant, 1985; Miceli et al., 1990; Atoji et al., 2018; Atoji and Wild, 2019). Despite some species differences, *5-Htr1F* is expressed in the intermediate cortical layers (layers IV and V) in mammals (Waeber and Moskowitz 1995; Mengod et al., 1996; Pascual et al., 1996; Lucaites et al., 2005). Taken together, both *5-HTR1F*-expressing neurons in chick IHA and *5-Htr1F*-expressing neurons in layer IV of the mammalian neocortex might have conserved functions in processing sensory input under serotonergic modulation.

We also detected that *5-HTR5A* was expressed in the dorsal arcopallium, lateral nidopallium, HF, and CDL. In mammalian brains, *5-HTR5A* is distributed in the piriform cortex, habenula, and HF, suggesting its involvement in the regulation of cognition, anxiety, and sensory perception (Plassat et al., 1992; Erlander et al., 1993; Matthes et al., 1993; Kinsey et al., 2001; Mengod et al., 2006; Vilaro et al., 2020). The dorsal arcopallium is the proposed region homologous to the basolateral amygdala in terms of embryonic origin and expression combinations of conserved morphogenetic genes (Martinez-Garcia et al., 2009; Medina et al., 2017a; Martinez-Garcia and Lanuza, 2018). The similarity in the regional expression of *5-HTR5A* between mammalian and chick telencephalons suggested its conserved function in the serotonergic modulation in telencephalons.

Finally, we observed that *5-HTR7* was expressed in the arcopallium, lateral nidopallium, HF, and CDL. In the mammalian telencephalon, *5-Htr7* is distributed in some brain regions, including the HF and amygdala (Gustafson et al., 1996; Mengod et al., 1996; Martin-Cora and Pazos, 2004; Mengod et al., 2006; Tanaka et al., 2012). This finding suggested the conserved 5-HTR7-mediated serotonergic modulation in the amygdala and HF between birds and mammals.

## Conclusion

We comprehensively revealed the expression patterns of 5-HTR subfamily genes in the chick telencephalon and specifically found that *5-HTR1D*, *5-HTR1E*, *5-HTR5A*, and *5-HTR7* were expressed in chick HF. These receptors might be involved in the regulation of HF neural circuits that control cognitive and emotion-related functions in birds. In addition, we found that *5-HTR1B*, *5-HTR1D*, *5-HTR1E*, and *5-HTR3A* were expressed in HF in a subdivision- and layer-selective manner. Our findings can facilitate the improved understanding of the correspondence between the avian and mammalian HF.

## Data Availability

The original contributions presented in the study are included in the article/Supplementary Material, further inquiries can be directed to the corresponding author.

## References

[B1] Abellán A. Desfilis E. Medina L. (2014). Combinatorial Expression of Lef1, Lhx2, Lhx5, Lhx9, Lmo3, Lmo4, and Prox1 Helps to Identify Comparable Subdivisions in the Developing Hippocampal Formation of Mouse and Chicken. Front. Neuroanat. 8, 59. 10.3389/fnana.2014.00059 25071464 PMC4082316

[B2] Anacker C. Hen R. (2017). Adult Hippocampal Neurogenesis and Cognitive Flexibility - Linking Memory and Mood. Nat. Rev. Neurosci. 18, 335–346. 10.1038/nrn.2017.45 28469276 PMC6261347

[B3] Atoji Y. Sarkar S. Wild J. M. (2018). Differential Projections of the Densocellular and Intermediate Parts of the Hyperpallium in the pigeon (*Columba livia*). J. Comp. Neurol. 526, 146–165. 10.1002/cne.24328 28891049

[B4] Atoji Y. Sarkar S. Wild J. M. (2016). Proposed Homology of the Dorsomedial Subdivision and V-Shaped Layer of the Avian hippocampus to Ammon's Horn and Dentate Gyrus, Respectively. Hippocampus 26, 1608–1617. 10.1002/hipo.22660 27657725

[B5] Atoji Y. Wild J. M. (2006). Anatomy of the Avian Hippocampal Formation. Rev. Neurosciences 17, 3–15. 10.1515/revneuro.2006.17.1-2.3 16703939

[B6] Atoji Y. Wild J. M. (2004). Fiber Connections of the Hippocampal Formation and Septum and Subdivisions of the Hippocampal Formation in the pigeon as Revealed by Tract Tracing and Kainic Acid Lesions. J. Comp. Neurol. 475, 426–461. 10.1002/cne.20186 15221956

[B7] Atoji Y. Wild J. M. (2019). Projections of the Densocellular Part of the Hyperpallium in the Rostral Wulst of Pigeons (*Columba livia*). Brain Res. 1711, 130–139. 10.1016/j.brainres.2019.01.001 30610876

[B8] Bacqué-Cazenave J. Bharatiya R. Barrière G. Delbecque J. P. Bouguiyoud N. Di Giovanni G. (2020). Serotonin in Animal Cognition and Behavior. Int. J. Mol. Sci. 21, 1649. 10.3390/ijms21051649 PMC7084567 32121267

[B9] Bagnoli P. Burkhalter A. (1983). Organization of the Afferent Projections to the Wulst in the pigeon. J. Comp. Neurol. 214, 103–113. 10.1002/cne.902140111 6841672

[B10] Belgard T. G. Montiel J. F. Wang W. Z. García-Moreno F. Margulies E. H. Ponting C. P. (2013). Adult Pallium Transcriptomes surprise in Not Reflecting Predicted Homologies across Diverse Chicken and Mouse Pallial Sectors. Proc. Natl. Acad. Sci. U.S.A. 110, 13150–13155. 10.1073/pnas.1307444110 23878249 PMC3740902

[B11] Bingman V. P. Gagliardo A. Hough G. E. Ioale P. Kahn M. C. Siegel J. J. (2005). The Avian hippocampus, Homing in Pigeons and the Memory Representation of Large-Scale Space. Integr. Comp. Biol. 45, 555–564. 10.1093/icb/45.3.555 21676801

[B12] Bocchio M. Mchugh S. B. Bannerman D. M. Sharp T. Capogna M. (2016). Serotonin, Amygdala and Fear: Assembling the Puzzle. Front. Neural Circuits 10, 24. 10.3389/fncir.2016.00024 27092057 PMC4820447

[B13] Bombardi C. (2014). Neuronal Localization of the 5-HT2 Receptor Family in the Amygdaloid Complex. Front. Pharmacol. 5, 68. 10.3389/fphar.2014.00068 24782772 PMC3988395

[B14] Bonaventure P. Guo H. Tian B. Liu X. Bittner A. Roland B. (2002). Nuclei and Subnuclei Gene Expression Profiling in Mammalian Brain. Brain Res. 943, 38–47. 10.1016/s0006-8993(02)02504-0 12088837

[B15] Bruinvels A. T. Landwehrmeyer B. Gustafson E. L. Durkin M. M. Mengod G. Branchek T. A. (1994a). Localization of 5-HT1B, 5-HT1Dα, 5-HT1E and 5-HT1F Receptor Messenger RNA in Rodent and Primate Brain. Neuropharmacology 33, 367–386. 10.1016/0028-3908(94)90067-1 7984275

[B16] Bruinvels A. T. Landwehrmeyer B. Probst A. Palacios J. Hoyer D. (1994b). A Comparative Autoradiographic Study of 5-HT1D Binding Sites in Human and guinea-pig Brain Using Different Radioligands. Mol. Brain Res. 21, 19–29. 10.1016/0169-328x(94)90374-3 8164519

[B17] Colombo M. Broadbent N. (2000). Is the Avian hippocampus a Functional Homologue of the Mammalian hippocampus? Neurosci. Biobehavioral Rev. 24, 465–484. 10.1016/s0149-7634(00)00016-6 10817844

[B18] Corrales Parada C. D. Morandi-Raikova A. Rosa-Salva O. Mayer U. (2021). Neural Basis of Unfamiliar Conspecific Recognition in Domestic Chicks (Gallus *Gallus domesticus*). Behav. Brain Res. 397, 112927. 10.1016/j.bbr.2020.112927 32980353

[B19] D. Székely A. (1999). The Avian Hippocampal Formation: Subdivisions and Connectivity. Behav. Brain Res. 98, 219–225. 10.1016/s0166-4328(98)00087-4 10683110

[B20] Erlander M. G. Lovenberg T. W. Baron B. M. De Lecea L. Danielson P. E. Racke M. (1993). Two Members of a Distinct Subfamily of 5-hydroxytryptamine Receptors Differentially Expressed in Rat Brain. Proc. Natl. Acad. Sci. U.S.A. 90, 3452–3456. 10.1073/pnas.90.8.3452 7682702 PMC46318

[B21] Faber H. Braun K. Zuschratter W. Scheich H. (1989). System-specific Distribution of Zinc in the Chick Brain. A Light- and Electron-Microscopic Study Using the Timm Method. Cell Tissue Res 258, 247–257. 10.1007/BF00239445 2582476

[B22] Fanselow M. S. Dong H.-W. (2010). Are the Dorsal and Ventral Hippocampus Functionally Distinct Structures? Neuron 65, 7–19. 10.1016/j.neuron.2009.11.031 20152109 PMC2822727

[B23] Fernandez A. S. Pieau C. Reperant J. Boncinelli E. Wassef M. (1998). Expression of the Emx-1 and Dlx-1 Homeobox Genes Define Three Molecularly Distinct Domains in the Telencephalon of Mouse, Chick, Turtle and Frog Embryos: Implications for the Evolution of Telencephalic Subdivisions in Amniotes. Development 125, 2099–2111. 10.1242/dev.125.11.2099 9570774

[B24] Fonseca M. I. Ni Y. G. Dunning D. D. Miledi R. (2001). Distribution of Serotonin 2A, 2C and 3 Receptor mRNA in Spinal Cord and Medulla Oblongata. Mol. Brain Res. 89, 11–19. 10.1016/s0169-328x(01)00049-3 11311971

[B25] Fujita T. Aoki N. Fujita E. Matsushima T. Homma K. J. Yamaguchi S. (2019). The Chick Pallium Displays Divergent Expression Patterns of Chick Orthologues of Mammalian Neocortical Deep Layer-specific Genes. Sci. Rep. 9, 20400. 10.1038/s41598-019-56960-4 31892722 PMC6938507

[B26] Fujita T. Aoki N. Mori C. Fujita E. Matsushima T. Homma K. J. (2020). The Dorsal Arcopallium of Chicks Displays the Expression of Orthologs of Mammalian Fear Related Serotonin Receptor Subfamily Genes. Sci. Rep. 10, 21183. 10.1038/s41598-020-78247-9 33273690 PMC7712838

[B27] Fujita T. Aoki N. Mori C. Fujita E. Matsushima T. Homma K. J. (2022). Serotonergic Neurons in the Chick Brainstem Express Various Serotonin Receptor Subfamily Genes. Front. Physiol. 2548, 815997. 10.3389/fphys.2021.815997 PMC8801614 35111079

[B28] Galceran J. Miyashita-Lin E. M. Devaney E. Rubenstein J. L. Grosschedl R. (2000). Hippocampus Development and Generation of Dentate Gyrus Granule Cells Is Regulated by LEF1. Development 127, 469–482. 10.1242/dev.127.3.469 10631168

[B29] Gupta S. Maurya R. Saxena M. Sen J. (2012). Defining Structural Homology between the Mammalian and Avian hippocampus through Conserved Gene Expression Patterns Observed in the Chick Embryo. Dev. Biol. 366, 125–141. 10.1016/j.ydbio.2012.03.027 22537492

[B30] Gustafson E. L. Durkin M. M. Bard J. A. Zgombick J. Branchek T. A. (1996). A Receptor Autoradiographic and *In Situ* Hybridization Analysis of the Distribution of the 5-ht7 Receptor in Rat Brain. Br. J. Pharmacol. 117, 657–666. 10.1111/j.1476-5381.1996.tb15241.x 8646411 PMC1909328

[B31] Hanics J. Teleki G. Alpár A. Székely A. D. Csillag A. (2017). Multiple Amygdaloid Divisions of Arcopallium Send Convergent Projections to the Nucleus Accumbens and Neighboring Subpallial Amygdala Regions in the Domestic Chicken: a Selective Pathway Tracing and Reconstruction Study. Brain Struct. Funct. 222, 301–315. 10.1007/s00429-016-1219-8 27053075 PMC5225175

[B32] Herold C. Bingman V. P. Ströckens F. Letzner S. Sauvage M. Palomero-Gallagher N. (2014). Distribution of Neurotransmitter Receptors and Zinc in the Pigeon (*Columba livia*) Hippocampal Formation: A Basis for Further Comparison with the Mammalian Hippocampus. J. Comp. Neurol. 522, 2553–2575. 10.1002/cne.23549 24477871

[B33] Herold C. Coppola V. J. Bingman V. P. (2015). The Maturation of Research into the Avian Hippocampal Formation: Recent Discoveries from One of the Nature's Foremost Navigators. Hippocampus 25, 1193–1211. 10.1002/hipo.22463 25850561

[B34] Herold C. Schlömer P. Mafoppa-Fomat I. Mehlhorn J. Amunts K. Axer M. (2019). The hippocampus of Birds in a View of Evolutionary Connectomics. Cortex 118, 165–187. 10.1016/j.cortex.2018.09.025 30442359

[B35] Hevner R. F. (2016). Evolution of the Mammalian Dentate Gyrus. J. Comp. Neurol. 524, 578–594. 10.1002/cne.23851 26179319 PMC4706817

[B36] Huang Y.-Y. Kandel E. R. (2007). 5-hydroxytryptamine Induces a Protein Kinase A/mitogen-activated Protein Kinase-Mediated and Macromolecular Synthesis-dependent Late Phase of Long-Term Potentiation in the Amygdala. J. Neurosci. 27, 3111–3119. 10.1523/jneurosci.3908-06.2007 17376972 PMC6672482

[B37] Ikebuchi M. Hasegawa T. Bischof H.-J. (2009). Amygdala and Socio-Sexual Behavior in Male Zebra Finches. Brain Behav. Evol. 74, 250–257. 10.1159/000264660 19996583

[B38] International Chicken Genome Sequencing Consortium (2004). Sequence and Comparative Analysis of the Chicken Genome Provide Unique Perspectives on Vertebrate Evolution. Nature 432, 695–716. 10.1038/nature03154 15592404

[B39] Jarvis E. D. Yu J. Rivas M. V. Horita H. Feenders G. Whitney O. (2013). Global View of the Functional Molecular Organization of the Avian Cerebrum: Mirror Images and Functional Columns. J. Comp. Neurol. 521, 3614–3665. 10.1002/cne.23404 23818122 PMC4145244

[B40] Kandel E. R. Schwartz J. H. Jessell T. M. Siegelbaum S. Hudspeth A. J. Mack S. (2000). Principles of Neural Science. New York: McGraw-Hill.

[B41] Kempermann G. (2012). New Neurons for 'survival of the Fittest'. Nat. Rev. Neurosci. 13, 727–736. 10.1038/nrn3319 22948073

[B42] Kinsey A. M. Wainwright A. Heavens R. Sirinathsinghji D. J. S. Oliver K. R. (2001). Distribution of 5-HT5A, 5-HT5B, 5-HT6 and 5-HT7 Receptor mRNAs in the Rat Brain. Mol. Brain Res. 88, 194–198. 10.1016/s0169-328x(01)00034-1 11295248

[B43] Koyama Y. Kondo M. Shimada S. (2017). Building a 5-HT3A Receptor Expression Map in the Mouse Brain. Sci. Rep. 7, 42884. 10.1038/srep42884 28276429 PMC5343592

[B44] Krause E. T. Kjaer J. B. Dudde A. Schrader L. Phi-Van L. (2019). Fear but Not Social Behaviour Is Affected by a Polymorphism in the 5'-flanking Region of the Serotonin Transporter (5-HTT) Gene in Adult Hens. Behav. Brain Res. 361, 50–53. 10.1016/j.bbr.2018.12.029 30562569

[B45] Krause E. T. Kjaer J. B. Lüders C. Van L. P. (2017). A Polymorphism in the 5′-flanking Region of the Serotonin Transporter (5-HTT) Gene Affects Fear-Related Behaviors of Adult Domestic Chickens. Behav. Brain Res. 330, 92–96. 10.1016/j.bbr.2017.04.051 28465138

[B46] Kuenzel W. J. Masson M. (1988). A Stereotaxic Atlas of the Brain of the Chick (*Gallus domesticus*). Baltimore: Johns Hopkins University Press.

[B47] Lowther S. Paermentier F. D. Crompton M. R. Horton R. W. (1992). The Distribution of 5-HT1D and 5-HT1E Binding Sites in Human Brain. Eur. J. Pharmacol. 222, 137–142. 10.1016/0014-2999(92)90473-h 1468490

[B48] Lucaites V. L. Krushinski J. H. Schaus J. M. Audia J. E. Nelson D. L. (2005). [3H]LY334370, a Novel Radioligand for the 5-HT1F Receptor. II. Autoradiographic Localization in Rat, guinea Pig, Monkey and Human Brain. Naunyn-schmiedeberg's Arch. Pharmacol. 371, 178–184. 10.1007/s00210-005-1036-8 15900511

[B49] Luo X. Muñoz-Pino E. Francavilla R. Vallée M. Droit A. Topolnik L. (2019). Transcriptomic Profile of the Subiculum-Projecting VIP GABAergic Neurons in the Mouse CA1 hippocampus. Brain Struct. Funct. 224, 2269–2280. 10.1007/s00429-019-01883-z 31098764

[B50] Marin P. Bécamel C. Chaumont-Dubel S. Vandermoere F. Bockaert J. Claeysen S. (2020). “Classification and Signaling Characteristics of 5-HT Receptors: toward the Concept of 5-HT Receptosomes,” in Handbook of Behavioral Neuroscience (Elsevier), 91–120. 10.1016/b978-0-444-64125-0.00005-0

[B51] Martín‐Cora F. J. Pazos A. (2004). Autoradiographic Distribution of 5‐HT7 Receptors in the Human Brain Using [3H] Mesulergine: Comparison to Other Mammalian Species. Br. J. Pharmacol. 141, 92–104. 10.1038/sj.bjp.0705576 14656806 PMC1574165

[B52] Martínez-García F. Lanuza E. (2018). Evolution of Vertebrate Survival Circuits. Curr. Opin. Behav. Sci. 24, 113–123. 10.1016/j.cobeha.2018.06.012

[B53] Martınez-Garcıa F. Novejarque A. Lanuza E. (2009). 15 the Evolution of the Amygdala in Vertebrates. Evol. Neurosci. 313, 392. 10.1016/B0-12-370878-8/00139-7

[B54] Matsushima T. Izawa E.-I. Aoki N. Yanagihara S. (2003). The Mind through Chick Eyes : Memory, Cognition and Anticipation. Zoolog. Sci. 20, 395–408. 10.2108/zsj.20.395 12719641

[B55] Matthes H. Boschert U. Amlaiky N. Grailhe R. Plassat J. L. Muscatelli F. (1993). Mouse 5-hydroxytryptamine5A and 5-hydroxytryptamine5B Receptors Define a New Family of Serotonin Receptors: Cloning, Functional Expression, and Chromosomal Localization. Mol. Pharmacol. 43, 313–319. 8450829

[B56] Mayer U. Rosa-Salva O. Loveland J. L. Vallortigara G. (2019). Selective Response of the Nucleus Taeniae of the Amygdala to a Naturalistic Social Stimulus in Visually Naive Domestic Chicks. Sci. Rep. 9, 9849. 10.1038/s41598-019-46322-5 31285532 PMC6614359

[B57] Mayer U. Bhushan R. Vallortigara G. Lee S. A. (2018). Representation of Environmental Shape in the hippocampus of Domestic Chicks (Gallus gallus). Brain Struct. Funct. 223, 941–953. 10.1007/s00429-017-1537-5 29032391

[B58] Mayer U. Pecchia T. Bingman V. P. Flore M. Vallortigara G. (2016). Hippocampus and Medial Striatum Dissociation during Goal Navigation by Geometry or Features in the Domestic Chick: An Immediate Early Gene Study. Hippocampus 26, 27–40. 10.1002/hipo.22486 26135386

[B59] Mayer U. Rosa-Salva O. Vallortigara G. (2017). First Exposure to an Alive Conspecific Activates Septal and Amygdaloid Nuclei in Visually-Naïve Domestic Chicks (Gallus gallus). Behav. Brain Res. 317, 71–81. 10.1016/j.bbr.2016.09.031 27633562

[B60] Medina L. Abellán A. Desfilis E. (2017b). Contribution of Genoarchitecture to Understanding Hippocampal Evolution and Development. Brain Behav. Evol. 90, 25–40. 10.1159/000477558 28866679

[B61] Medina L. Abellán A. Vicario A. Castro-Robles B. Desfilis E. (2017a). “The Amygdala,” in Evolution of Nervous Systems (Second Edition). 1, 427–478. 10.1016/B978-0-12-804042-3.00019-1

[B62] Medina L. Reiner A. (2000). Do birds Possess Homologues of Mammalian Primary Visual, Somatosensory and Motor Cortices? Trends Neurosciences 23, 1–12. 10.1016/s0166-2236(99)01486-1 10631781

[B63] Mengod G. Vilaró M. T. Cortés R. López-Giménez J. F. Raurich A. Palacios J. M. (2006). “Chemical Neuroanatomy of 5-HT Receptor Subtypes in the Mammalian Brain,” in The serotonin receptors (Humana Press). Editors Roth B. L., 319–364. 10.1007/978-1-59745-080-5_10

[B64] Mengod G. Vilaró M. T. Raurich A. López-Giménez J. F. Cortés R. Palacios J. M. (1996). 5-HT Receptors in Mammalian Brain: Receptor Autoradiography Andin Situ Hybridization Studies of New Ligands and Newly Identified Receptors. Histochem. J. 28, 747–758. 10.1007/bf02272148 8968727

[B65] Miceli D. Repérant J. (1985). Telencephalic Afferent Projections from the Diencephalon and Brainstem in the pigeon. A Retrograde Multiple-Label Fluorescent Study. Exp. Biol. 44, 71–99. 3850028

[B66] Miceli D. Marchand L. Repérant J. Rio J.-P. (1990). Projections of the Dorsolateral Anterior Complex and Adjacent Thalamic Nuclei upon the Visual Wulst in the pigeon. Brain Res. 518, 317–323. 10.1016/0006-8993(90)90990-s 1697211

[B67] Montagnese C. M. Geneser F. A. Krebs J. R. (1993). Histochemical Distribution of Zinc in the Brain of the Zebra Finch (Taenopygia Guttata). Anat. Embryol. (Berl) 188, 173–187. 10.1007/BF00186251 8214632

[B68] Montagnese C. M. Krebs J. R. Meyer G. (1996). The Dorsomedial and Dorsolateral Forebrain of the Zebra Finch, *Taeniopygia guttata*: ;A Golgi Study. Cel Tissue Res. 283, 263–282. 10.1007/s004410050537 8593656

[B69] Morales M. Wang S.-D. (2002). Differential Composition of 5-Hydroxytryptamine3Receptors Synthesized in the Rat CNS and Peripheral Nervous System. J. Neurosci. 22, 6732–6741. 10.1523/jneurosci.22-15-06732.2002 12151552 PMC6758137

[B70] Morandi-Raikova A. Mayer U. (2020). The Effect of Monocular Occlusion on Hippocampal C-Fos Expression in Domestic Chicks (Gallus gallus). Sci. Rep. 10, 7205. 10.1038/s41598-020-64224-9 32350337 PMC7190859

[B71] O'leary O. F. Codagnone M. G. Cryan J. F. (2020). “Revisiting the Behavioral Genetics of Serotonin: Relevance to Anxiety and Depression,” in Handbook of Behavioral Neuroscience (Elsevier), 665–709. 10.1016/b978-0-444-64125-0.00038-4

[B72] Papini M. R. Penagos-Corzo J. C. Pérez-Acosta A. M. (2019). Avian Emotions: Comparative Perspectives on Fear and Frustration. Front. Psychol. 9, 2707. 10.3389/fpsyg.2018.02707 30705652 PMC6344452

[B73] Pascual J. Del Arco C. Romón T. Del Olmo E. Pazos A. (1996). [3H]Sumatriptan Binding Sites in Human Brain: Regional-dependent Labelling of 5-HT1D and 5-HT1F Receptors. Eur. J. Pharmacol. 295, 271–274. 10.1016/0014-2999(95)00748-2 8720594

[B74] Payne H. L. Lynch G. F. Aronov D. (2021). Neural Representations of Space in the hippocampus of a Food-Caching Bird. Science 373 **,** 343–348. 10.1126/science.abg2009 34437154 PMC8503942

[B75] Phi Van V. D. Krause E. T. Phi-Van L. (2018). Modulation of Fear and Arousal Behavior by Serotonin Transporter (5-HTT) Genotypes in Newly Hatched Chickens. Front. Behav. Neurosci. 12, 284. 10.3389/fnbeh.2018.00284 30524254 PMC6256247

[B76] Plassat J. L. Boschert U. Amlaiky N. Hen R. (1992). The Mouse 5HT5 Receptor Reveals a Remarkable Heterogeneity within the 5HT1D Receptor Family. EMBO J. 11, 4779–4786. 10.1002/j.1460-2075.1992.tb05583.x 1464308 PMC556953

[B77] Puelles L. Kuwana E. Puelles E. Bulfone A. Shimamura K. Keleher J. (2000). Pallial and Subpallial Derivatives in the Embryonic Chick and Mouse Telencephalon, Traced by the Expression of the Genes Dlx-2, Emx-1, Nkx-2.1, Pax-6, and Tbr-1. J. Comp. Neurol. 424, 409–438. 10.1002/1096-9861(20000828)424:3<409::aid-cne3>3.0.co;2-7 10906711

[B78] Puelles L. Martinez-de-la-Torre M. Watson C. Martinez S. Paxinos G. (2007). The Chick Brain in Stereotaxic Coordinates and Alternate Stains. San Diego: Academic Press, Elsevier.

[B79] Redies C. Medina L. Puelles L. (2001). Cadherin Expression by Embryonic Divisions and Derived gray Matter Structures in the Telencephalon of the Chicken. J. Comp. Neurol. 438, 253–285. 10.1002/cne.1315 11550172

[B80] Reiner A. Perkel D. J. Bruce L. L. Butler A. B. Csillag A. Kuenzel W. (2004). Revised Nomenclature for Avian Telencephalon and Some Related Brainstem Nuclei. J. Comp. Neurol. 473, 377–414. 10.1002/cne.20118 15116397 PMC2518311

[B81] Rosa Salva O. Mayer U. Vallortigara G. (2015). Roots of a Social Brain: Developmental Models of Emerging Animacy-Detection Mechanisms. Neurosci. Biobehavioral Rev. 50, 150–168. 10.1016/j.neubiorev.2014.12.015 25544151

[B82] Sherry D. F. Grella S. L. Guigueno M. F. White D. J. Marrone D. F. (2017). Are There Place Cells in the Avian Hippocampus? Brain Behav. Evol. 90, 73–80. 10.1159/000477085 28866682

[B83] Sloviter R. S. Lømo T. (2012). Updating the Lamellar Hypothesis of Hippocampal Organization. Front. Neural Circuits 6, 102. 10.3389/fncir.2012.00102 23233836 PMC3517983

[B84] Smulders T. V. (2021). Telencephalic Regulation of the HPA axis in Birds. Neurobiol. Stress 15, 100351. 10.1016/j.ynstr.2021.100351 34189191 PMC8220096

[B85] Smulders T. V. (2017). The Avian Hippocampal Formation and the Stress Response. Brain Behav. Evol. 90, 81–91. 10.1159/000477654 28866683

[B86] Strac D. S. Pivac N. Muck-Seler D. (2016). The Serotonergic System and Cognitive Function. Translational Neurosci. 7, 35–49. 10.1515/tnsci-2016-0007 PMC5017596 28123820

[B87] Striedter G. F. (2016). Evolution of the Hippocampus in Reptiles and Birds. J. Comp. Neurol. 524, 496–517. 10.1002/cne.23803 25982694

[B88] Suárez J. Dávila J. C. Real M. á. Guirado S. Medina L. (2006). Calcium-binding Proteins, Neuronal Nitric Oxide Synthase, and GABA Help to Distinguish Different Pallial Areas in the Developing and Adult Chicken. I. Hippocampal Formation and Hyperpallium. J. Comp. Neurol. 497, 751–771. 10.1002/cne.21004 16786551

[B89] Tanaka K. F. Samuels B. A. Hen R. (2012). Serotonin Receptor Expression along the Dorsal-Ventral axis of Mouse hippocampus. Phil. Trans. R. Soc. B 367, 2395–2401. 10.1098/rstb.2012.0038 22826340 PMC3405677

[B90] Tecott L. H. Maricq A. V. Julius D. (1993). Nervous System Distribution of the Serotonin 5-HT3 Receptor mRNA. Proc. Natl. Acad. Sci. U.S.A. 90, 1430–1434. 10.1073/pnas.90.4.1430 8434003 PMC45887

[B91] Tömböl T. Davies D. C. Németh A. Sebestény T. Alpár A. (2000). A Comparative Golgi Study of Chicken (*Gallus domesticus*) and Homing pigeon (*Columba livia*) hippocampus. Anat. Embryol. 201, 85–101. 10.1007/pl00008235 10672361

[B92] Tosches M. A. Yamawaki T. M. Naumann R. K. Jacobi A. A. Tushev G. Laurent G. (2018). Evolution of Pallium, hippocampus, and Cortical Cell Types Revealed by Single-Cell Transcriptomics in Reptiles. Science 360, 881–888. 10.1126/science.aar4237 29724907

[B93] Vilaró M. Cortés R. Mengod G. Hoyer D. (2020). “Distribution of 5-HT Receptors in the central Nervous System: an Update,” in Handbook of Behavioral Neuroscience (Elsevier), 121–146.

[B94] Waeber C. Moskowitz M. A. (1995). [3H]sumatriptan Labels Both 5-HT1D and 5-HT1F Receptor Binding Sites in the guinea Pig Brain: an Autoradiographic Study. Naunyn Schmiedebergs Arch. Pharmacol. 352, 263–275. 10.1007/BF00168556 8584041

[B95] Yamaguchi S. Fujii-Taira I. Katagiri S. Izawa E.-I. Fujimoto Y. Takeuchi H. (2008a). Gene Expression Profile in Cerebrum in the Filial Imprinting of Domestic Chicks (Gallus gallus Domesticus). Brain Res. Bull. 76, 275–281. 10.1016/j.brainresbull.2008.02.002 18498941

[B96] Yamaguchi S. Fujii-Taira I. Murakami A. Hirose N. Aoki N. Izawa E.-I. (2008b). Up-regulation of Microtubule-Associated Protein 2 Accompanying the Filial Imprinting of Domestic Chicks (Gallus gallus Domesticus). Brain Res. Bull. 76, 282–288. 10.1016/j.brainresbull.2008.02.010 18498942

[B97] Yamamoto K. Reiner A. (2005). Distribution of the Limbic System-Associated Membrane Protein (LAMP) in pigeon Forebrain and Midbrain. J. Comp. Neurol. 486, 221–242. 10.1002/cne.20562 15844168

[B98] Yamamoto K. Sun Z. Wang H. B. Reiner A. (2005). Subpallial Amygdala and Nucleus Taeniae in Birds Resemble Extended Amygdala and Medial Amygdala in Mammals in Their Expression of Markers of Regional Identity. Brain Res. Bull. 66, 341–347. 10.1016/j.brainresbull.2005.02.016 16144611

[B99] Zmudzka E. Salaciak K. Sapa J. Pytka K. (2018). Serotonin Receptors in Depression and Anxiety: Insights from Animal Studies. Life Sci. 210, 106–124. 10.1016/j.lfs.2018.08.050 30144453

