# Supplementary material for: Chick Hippocampal Formation Displays Subdivision- and Layer-Selective Expression Patterns of Serotonin Receptor Subfamily Genes
Source: Front Physiol. 2022 Apr 8;13:882633. doi: 10.3389/fphys.2022.882633 (PMC9024137; doi:10.3389/fphys.2022.882633)
Supplement: Supplementary file 1 [file Presentation1.PPTX]

## Slide 1
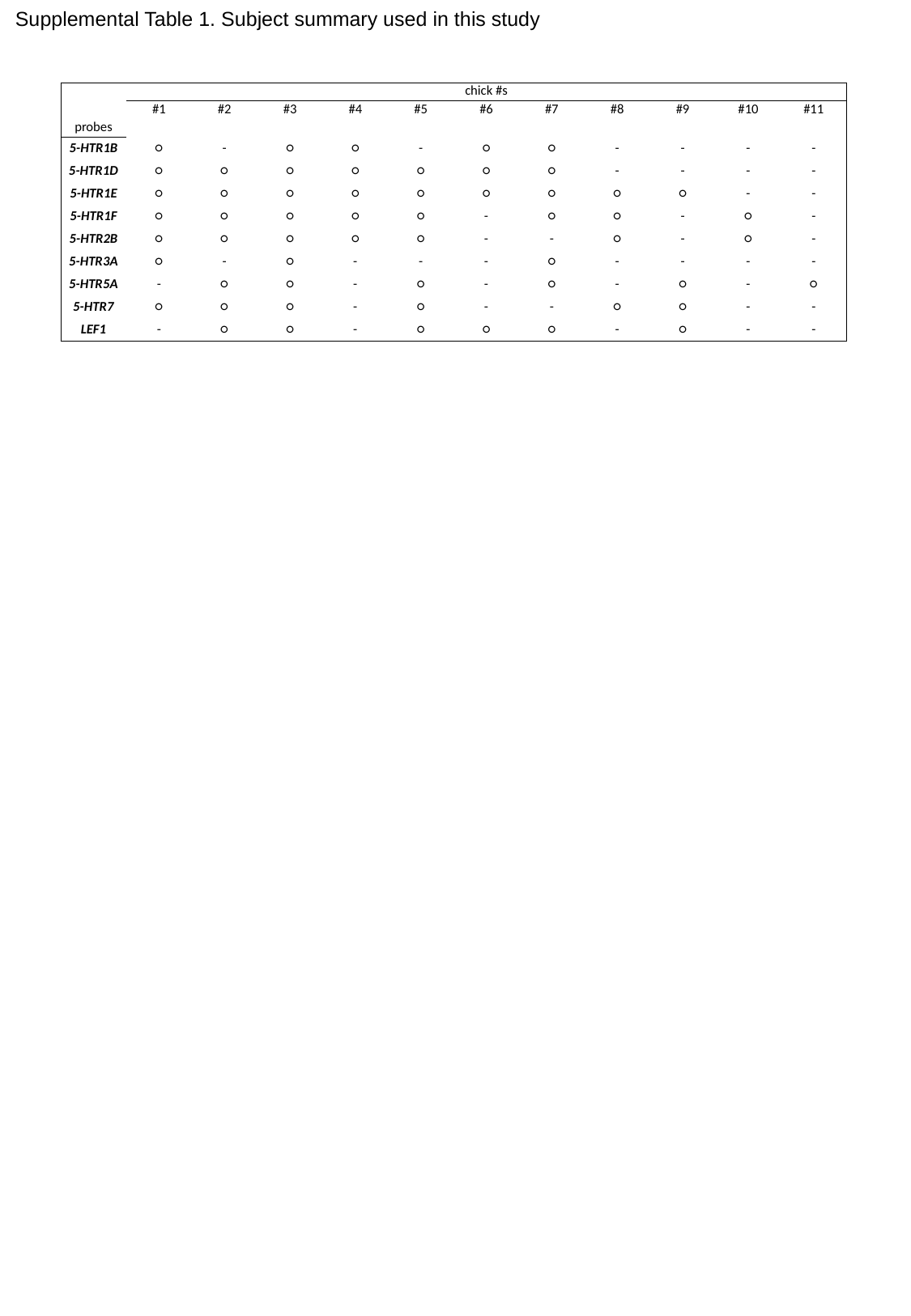

Supplemental Table 1. Subject summary used in this study
| | chick #s | | | | | | | | | | |
| --- | --- | --- | --- | --- | --- | --- | --- | --- | --- | --- | --- |
| | #1 | #2 | #3 | #4 | #5 | #6 | #7 | #8 | #9 | #10 | #11 |
| probes | | | | | | | | | | | |
| 5-HTR1B | ○ | - | ○ | ○ | - | ○ | ○ | - | - | - | - |
| 5-HTR1D | ○ | ○ | ○ | ○ | ○ | ○ | ○ | - | - | - | - |
| 5-HTR1E | ○ | ○ | ○ | ○ | ○ | ○ | ○ | ○ | ○ | - | - |
| 5-HTR1F | ○ | ○ | ○ | ○ | ○ | - | ○ | ○ | - | ○ | - |
| 5-HTR2B | ○ | ○ | ○ | ○ | ○ | - | - | ○ | - | ○ | - |
| 5-HTR3A | ○ | - | ○ | - | - | - | ○ | - | - | - | - |
| 5-HTR5A | - | ○ | ○ | - | ○ | - | ○ | - | ○ | - | ○ |
| 5-HTR7 | ○ | ○ | ○ | - | ○ | - | - | ○ | ○ | - | - |
| LEF1 | - | ○ | ○ | - | ○ | ○ | ○ | - | ○ | - | - |

## Slide 2
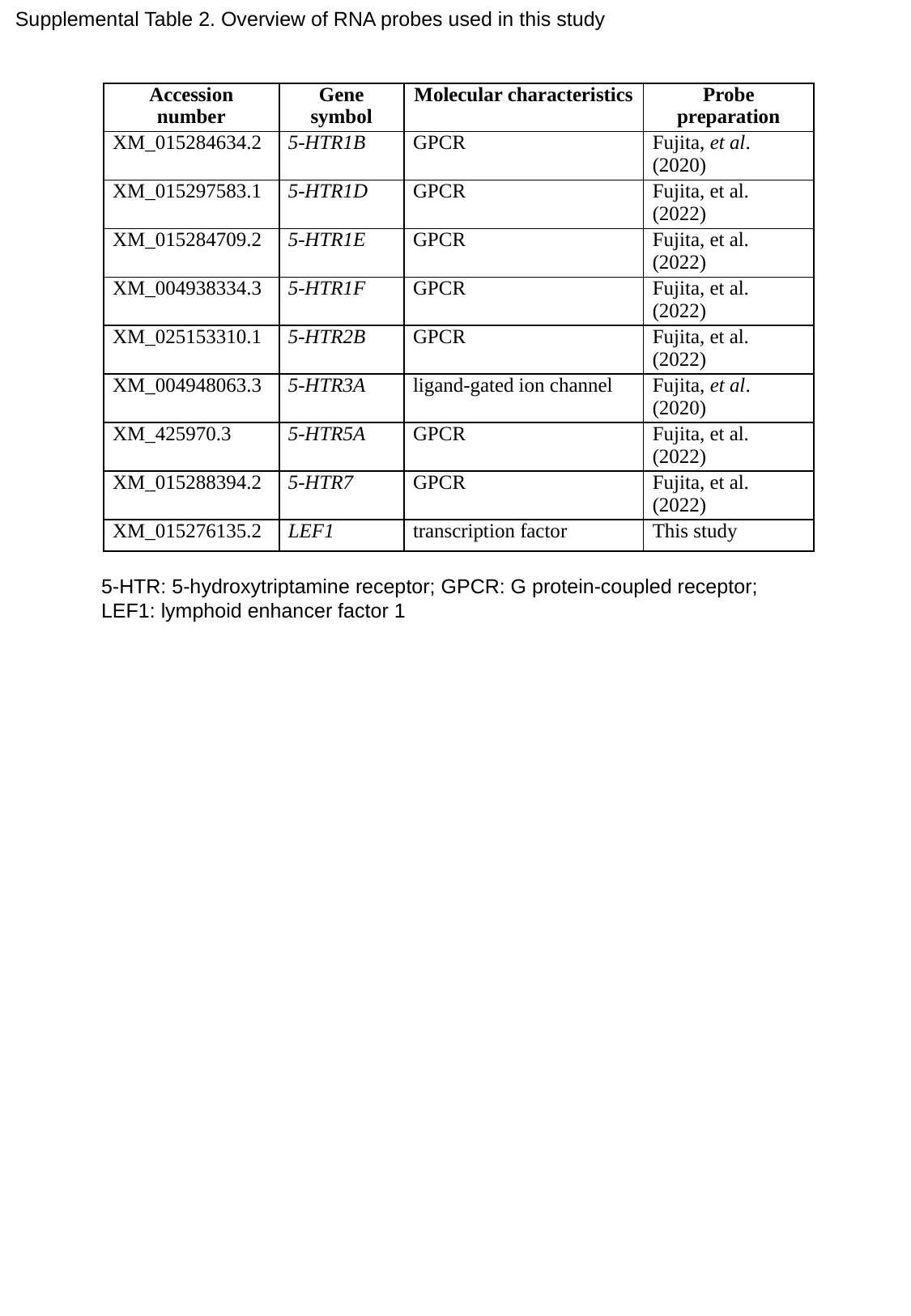

Supplemental Table 2. Overview of RNA probes used in this study
| Accession number | Gene symbol | Molecular characteristics | Probe preparation |
| --- | --- | --- | --- |
| XM\_015284634.2 | 5-HTR1B | GPCR | Fujita, et al. (2020) |
| XM\_015297583.1 | 5-HTR1D | GPCR | Fujita, et al. (2022) |
| XM\_015284709.2 | 5-HTR1E | GPCR | Fujita, et al. (2022) |
| XM\_004938334.3 | 5-HTR1F | GPCR | Fujita, et al. (2022) |
| XM\_025153310.1 | 5-HTR2B | GPCR | Fujita, et al. (2022) |
| XM\_004948063.3 | 5-HTR3A | ligand-gated ion channel | Fujita, et al. (2020) |
| XM\_425970.3 | 5-HTR5A | GPCR | Fujita, et al. (2022) |
| XM\_015288394.2 | 5-HTR7 | GPCR | Fujita, et al. (2022) |
| XM\_015276135.2 | LEF1 | transcription factor | This study |
5-HTR: 5-hydroxytriptamine receptor; GPCR: G protein-coupled receptor; LEF1: lymphoid enhancer factor 1
